# Growth Performance of Gilthead Sea Bream (*Sparus aurata*) Fed Low Fish Meal Diets With an Innovative Mixture of Low Trophic Ingredients

**DOI:** 10.1155/anu/7504207

**Published:** 2025-07-26

**Authors:** Anna Tampou, Katerina Kousoulaki, Antigoni Vasilaki, Nikolaos Vlahos, Eleni Nikouli, Nikolas Panteli, Konstantinos Feidantsis, Konstantinos Kormas, Styliani Andreopoulou, Ioannis T. Karapanagiotidis, Panagiotis Berillis, Ioannis Nengas, Efthimia Antonopoulou, Eleni Mente

**Affiliations:** ^1^Department of Ichthyology and Aquatic Environment, School of Agricultural Sciences, University of Thessaly GR-38446, Volos, Greece; ^2^Department of Nutrition and Feed Technology, Nofima AS, Kjerreidviken 16, Fyllingsdalen N-5141, Norway; ^3^Institute of Marine Biology, Biotechnology and Aquaculture, Hellenic Center for Marine Research, Anavyssos GR-19013, Athens, Greece; ^4^Department of Fisheries and Aquaculture, School of Agricultural Sciences, University of Patras GR-26504, Messolonghi, Greece; ^5^Department of Zoology, School of Biology, Aristotle University of Thessaloniki GR-54124, Thessaloniki, Greece; ^6^School of Veterinary Medicine, Aristotle University of Thessaloniki GR-54124, Thessaloniki, Greece

**Keywords:** black soldier fly, gilthead sea bream, microalgae, tunicate

## Abstract

This study examined the growth performance, cellular signaling, and gut microbiome of gilthead sea bream (*Sparus aurata*) fed four diets using low-trophic-level ingredients for 65 days. Control (C) diet contained fish meal (FM) as the main protein source and fish oil (FO) as a lipid source. In the 0%FMFO diet all FM and FO present in the C diet was replaced with a combination of microalgae, insect meal (IM), and tunicate meal (TM). IM and TM diets were formulated to contain 20% of the novel protein ingredients, replacing 68.09% and 45.91% of FM in diet C, respectively. Compared to diet C, feed utilization and growth performance of *S*. *aurata* fed 0%FMFO diet was not different (*p* > 0.05) and TM diet had a significantly lower (*p*  < 0.05) specific growth rate (SGR), higher (*p*  < 0.05) feed conversion ratio (FCR) and decreased fillet lipid content (*p* < 0.05). In liver and foregut of fish fed TM diet the activity of lactate dehydrogenase (L-LDH) significant increased (*p* < 0.05), indicating higher glycolytic potential, whereas the decrease in Hsp70, Hsp90, AMPK, and p38 MAPK may indicate reduced stress response. Fish midgut microbiome included beneficial taxa for the host. The results suggested that the mixture of algae, insect, and TM could replace all FM and FO in gilthead sea bream diets without affecting the fish growth performance.

## 1. Introduction

The use of sustainably produced protein and lipid sources for the replacement of fish meal (FM) and fish oil (FO) in aquafeeds are in need to support future growth of the aquaculture sector. Alternative ingredients derived from low-trophic-level organisms such as bacteria, yeast, algae, and insects are currently receiving increasing attention. Microalgae biomasses can be environmentally friendly and are rich in essential fatty acids, amino acids, pigments, and vitamins [1, 2], and their use in aquafeeds can result in enhanced fish quality [3]. For instance, the diatom *Phaeodactylum tricornutum* contains high levels of eicosapentaenoic acid (EPA) [4], as well as phenolic compounds and carotenoids, which have been suggested to enhance fillet quality in salmon [5]. Use of *P*. *tricornutum* in diets for Senegalese sole larvae led to enhanced growth performance [6] and higher dietary nutrient digestibility in rainbow trout [7] and it has also been suggested as an effective replacement for dietary FO in gilthead sea bream larval diets [8, 9].

Microalgae species have been used as FO replacers in fish feeds to enhance the content of *n*−3 long chain polyunsaturated fatty acids (PUFAs) in white muscle [10]. The traustochytrid *Schizochytrium* sp., mainly referred to as microalgae, contain high levels of *n*−3 long chain PUFAs, especially docosahexaenoic acid (DHA) [11]. Dietary *Schizochytrium* sp. lipids are highly digestible [12, 13] and their use has led to enhanced PUFA concentration in the fillet and improved growth performance in *S*. *aurata* [14, 15], while leading to health benefits in other farmed fish species, such as rainbow trout and zebrafish [16, 17]. The combination of *Schizochytrium* sp. with other microalgae in the diets has shown positive effects on growth and fatty acid accumulation in *Pagrus major* [18] and *Sparus aurata* [19]. Katsoulis-Dimitriou et al. [20] showed that microalgae mixtures in sea bream diets can be utilized by the fish gut microbiota metabolizing fucose, potentially improving host metabolism.

Insects are among the most promising nutrient sources that may play a significant protein contributor to aquaculture feeds in the future. Several insects contain a high amount of functional ingredients such as chitin, proteins, fatty acids, and micronutrients [21, 22]. Insect meals (IMs) have been shown to be efficient for FM replacement without adverse effects on fish health [23–25] and have also been suggested to have immunomodulatory, antimicrobial, and antioxidant properties [26–29]. The ability of insects to bioconvert agricultural waste and nonfood ingredients into valuable biomass [26, 30, 31] may contribute to the compliance of food production with circular economy guidelines [32, 33]. Among the species that are considered as potential FM replacement candidates, *Hermetia illucens* is a highly promising insect with high content of proteins and fats, and a good amino acid profile, although methionine and lysine amounts present are lower than in the FM based protein sources [34–37]. Several studies have been conducted evaluating how *H. illucens* in the diet affects fish nutrition and health with controversial results [38, 39]. In *S*. *aurata* diet, *H*. *illucens* meal studies showed to have no effect on fish growth [25, 40, 41], enhance growth [42] or suppress it, depending on FM replacement and feed inclusion level [43–45].

Tunicates are low-trophic-level filter-feeding sea invertebrates that feed on algae-rich water and have the potential for utilization in fish feeds [46]. Their bodies contain, in dry matter, high levels of proteins (e.g., collagen), cellulose, and *n*−3 fatty acids as well as minerals [47] and bioactive compounds [48]. Such organism is *Ciona intestinalis*, which is rich in *n*−3 and *n*−6 PUFAs and has been suggested as an alternative ingredient for FO replacement [49]. Samuelsen et al. [50] reported that *C*. *intestinalis* meal, at 172 g/kg inclusion in feed, which replaced 64% of FM in the diet, resulted in pellet quality features without compromising the fat absorption capacity and fat leakage of the pellets following extrusion.

Availability and cost of raw materials are going to play a key role in FM replacement in industrial aqua-feed production in the future. Among the insects that could be used in fish feeds, *Musca domestica* appears to be commercially available, with the remaining insect species expected to increase according to demand [51]. The microalgae industry faces challenges in producing large quantities, while microalgal biomasses are not cost-effective, especially when further processed [52]. The cost of raw materials is expected to decrease due to future increased demand of novel ingredients, while the feed formulation has to be tailored made species specific. Another step to increase efficiency of novel ingredients, and therefore, the feed could be genetic selection of fish [53], which could also reduce feed costs.

Diet-associated changes have been demonstrated to ambiguously affect several aspects of fish development and health. In addition to their impact on fish growth [45, 50, 54], novel ingredients in aquafeeds have been reported to modulate both physiological status [55–57] and gut microbial communities [40, 58, 59]. Changes in cellular signaling and energy homeostasis, as well as the recruitment of molecular chaperones, occur during an altered nutrient status to facilitate adaptation to the dietary regime [60–64]. Furthermore, diet-induced stress may also be aggravated due to modifications in gut microbial communities, which contribute to changes in both microbial nutrient production and malabsorption [64, 65]. However, an adequate diet in essential nutrients favors the enrichment of beneficial host bacteria, thus, enhancing the diversity and multifaceted functionality of the gut microbiome [40, 66, 67]. IM in gilthead sea bream diet has controversial effects on fish growth [68] and defatted meal instead of full fat *H*. *illucens* meal is proven to have advantages in fish nutrition [44, 69, 70]. On the other hand, tunicate meal (TM) is a novel, alternative, and sustainable ingredient that has the potential to be suitable for FM replacement in the feed of *S*. *aurata*. The combination of TM with other known ingredients, such as insect and algae meals, may be the key to the total FM and FO replacement in fish feeds. Such a combination of low-trophic ingredients could be the future of the aquaculture feed industry, due to the ability of these organisms to be sustainably grown on agricultural wastes and be environmentally friendly. In this context, this study aimed to evaluate the global effect of FM and FO replacement with innovative ingredients, such as IM, TM, or their combination with algae meals, respectively, on growth performance, nutrient utilization, physiological status, gut microbiota, liver, and gut histology of gilthead sea bream (*S. aurata*).

## 2. Materials and Methods

### 2.1. Ethic's Approval

The experimental protocol was approved by the Ethics Committee of the Region of Thessaly, Veterinary Directorate, Department of Animal Protection-Medicines-Veterinary Applications (No. 18403/05-09-2019). The experimental procedures were performed at the Laboratory of Aquaculture, Department of Ichthyology and Aquatic Environment, University of Thessaly (EL-43BIO/exp-01). All experimental procedures were conducted according to the guidelines of EU Directive 2010/63/EU regarding the protection of animals used for scientific purposes and were applied by FELASA-accredited scientists (Functions A–D).

### 2.2. Diet Formulation

Four experimental diets were formulated to contain 15% lipids and 43% protein, though the analytical results revealed higher protein content in the control diet. The control diet (C) was designed to contain 26.55% FM from sustainable fisheries and 10.42% FO from sustainable fisheries, simulating the composition of a commercial diet. In the 0%FMFO diet, the FM and FO were completely replaced with a mixture of dry algae meal (*Phaeodactylum tricornutum*), dry thraustochytrid meal (*Schizochytrium limacinum*), defatted larval IM (*Hermetia illucens*), and dried TM (*Ciona intestinalis*). In the IM diet, 68.09% of the FM was replaced with defatted larval IM (*H. illucens*), and in the TM diet, the FM replacement was 45.91% with dried TM (*C. intestinalis*). Krill hydrolysate meal was added to the experimental feeds to counteract palatability issues. The formulation, proximate composition, amino acid profile, and fatty acid profile of the experimental diets are shown in Table 1.

### 2.3. Experimental Trial

The experimental trial was conducted at the aquaculture laboratory of the University of Thessaly, Greece. The acclimatization period for the groups of gilthead sea bream (*Sparus aurata*) juveniles used in the present trial was 15 days, where the fish were fed by hand with a commercial diet to apparent satiation. The initial mean weight of the fish was 6.57 ± 0.04 g. Fish (720 individuals in total) which were randomly distributed into 12 cylindrical tanks with 250 L volume each (60 fish per tank in triplicate treatments), that were part of three autonomous recirculating aquaculture systems. Each autonomous recirculating aquaculture system had four tanks (250 lL each) connected to an independent filter system, as described in Tampou et al. [71]. The fish, during the growth phase of the experiment, were fed three times per day (8:30, 14:00, and 17:00), 7 days per week, to apparent satiation by hand. The rearing water quality (20.9 ± 0.01°C, DO 6.76 ± 0.0 mg/L, pH 7.92 ± 0.0, *S* 30.56 ± 0.01 ppt, L:D 12:12) remained stable during the experiment. The experimental trial lasted 65 days, during which fish behavior and status were monitored before, during, and after every meal.

At the end of the experiment, fish were fasted for 24 h and all weighed individually under anesthesia to determine growth performance and feed utilization parameters. Eighteen gilthead sea bream individuals (4–6 fish for each analysis) per dietary treatment were euthanized with an overdose of tricaine methanesulfonate (MS 222, 300+ mg/L). The wet weights of the fish and fish liver were measured for the estimation of hepatosomatic index and the muscle was removed for nutrient profile analysis. The midgut section of *S*. *aurata* was aseptically separated and rinsed with sterile particle-free seawater (three times), without removing the digesta. For the intermediate metabolism enzymes analysis, the liver and foregut of fish were dissected and for western blot analysis, the liver of another four fish of each dietary group was taken. The tissues for microbiology, enzyme activity, and microbiology activity were rapidly stored in a freezer at −80°C until further analysis and muscle tissues were prepared for proximate composition analysis. Additionally, liver, posterior, and anterior gut samples were dissected from fish for histological analysis.

### 2.4. Chemical Composition Analysis in Feeds and White Muscle

Nutrient composition was assessed in the feeds and the white muscle (fillet) of gilthead sea bream. Protein determination in feeds and white muscle was conducted according to the Kjeldahl analysis (*N* × 6.25; Behr Labor-Technik GmbH, Germany). Lipids in feeds and fish fillet was analyzed by exhaustive Soxhlet extraction using petroleum ether (40–60°C, BP) in a Soxtherm Multistat/SX PC (Sox-416 Macro, Gerhard, Germany).

The gross energy content of feeds and white muscle was analyzed adiabatically using an IKA oxygen bomb calorimeter (C5000; IKA Werke GmbH, Staufen, Germany). Tissue and diet amino acid composition was determined as described by Lyndon et al. [72] and Mente et al. [73]. The essential and nonessential amino acid ratio (A/E) of each essential amino acid (EAA) and nonessential amino acid (NEAA) was calculated as the percentage of the total EAA and NEAA accordingly [74]. The fatty acid analysis of diets and white muscle samples was performed using the method described in Fountoulaki et al. [75].

### 2.5. Growth Performance, Nutrient Utilization, and Biometric Parameters

The estimation of growth performance and nutritional parameters was calculated using the following formulae:

- Weight gain (WG, g/fish) = Final body weight (g) − initial body weight (g)
- Feed intake (FI, g/fish) = Total feed consumed/number of fish
- Specific growth rate (SGR, %/day) = 100 x [ln (final body weight) − ln (initial body weight)] × days^−1^
- Feed conversion ratio (FCR) = FI (g) × wet WG^−1^ (g)
- Protein efficiency ratio (PER) = wet WG (g) × protein intake^−1^ (g)
- Lipid efficiency ratio (LER) = wet WG (g) × lipid intake^−1^ (g)
- Voluntary FI (VFI, %body weight/day) = 100 × total dry FI (g) × [(initial + final body weight (g)) × 0.5 × days]^−1^
- Survival (%) = 100 × number of fish at the end of the experiment × number of fish stocked^−1^
- Hepatosomatic index (%) = 100 × liver weight (g) × body weight^−1^ (g)

### 2.6. Intermediate Metabolism Enzymes

The activities of 3-hydroxyacyl CoA dehydrogenase (HOAD, EC 1.1.1.35.), lactate dehydrogenase (L-LDH, EC 1.1.1.27.) and citrate synthase (CS, EC 4.1.3.7.) in liver and foregut of seabream fed the control and the experimental diets were estimated, according to Driedzic and Almeida-Val [76]. The assays were performed at 18°C based on Moon and Mommsen [77], Sidell et al. [78], and Singer and Ballantyne [79]. The activities of HOAD and L-LDH were measured following by the oxidation of NADH at 340 nm (extinction coefficient = 6.22 L mol^−1^ cm^−1^) and CS activity was determined based on the reaction of free coenzyme A with DTNB at 12 nm (extinction coefficient = 13.6 L mol^−1^ cm^−1^). Enzyme activities are expressed as μmols of substrate min^−1^ g wet tissue^−1^.

### 2.7. Western Blot Analysis

The western blot analysis was performed as described by Antonopoulou et al. [80]. In brief, frozen liver tissues were homogenized and extracted on ice for 30 min in a cold lysis buffer, the protein concentration was determined in duplicate pool samples per dietary treatment using the Bio-Rad protein assay (Bio-Rad, Hercules, CA, USA) and proteins transferred electrophoretically onto nitrocellulose membranes. Nonspecific binding sites on the membranes were blocked with 5% (*w*/*v*) nonfat milk in TBST (tris buffered saline-Twin 20), at room temperature, for 30 min and the membranes were incubated overnight with the appropriate primary antibodies: monoclonal mouse anti-HSP70 (H5147, Sigma), monoclonal mouse anti-HSP90 (H1775, Sigma), polyclonal rabbit anti-phospho-p38 MAP kinase (9211, Cell Signaling, Beverly, MA, USA), polyclonal rabbit anti-p38 MAPK (9212, Cell Signaling, Beverly, MA, USA), monoclonal rabbit anti-phospho AMPK (2535, Cell Signaling, Beverly, MA, USA), monoclonal rabbit anti-AMPK (5831, Cell Signaling, Beverly, MA, USA), anti-phospho-Akt (9271, Cell Signaling, Beverly, MA, USA), and anti-Akt (9272, Cell Signaling, Beverly, MA, USA). The blots were washed with TBST (three periods, 5 min each time), incubated with horseradish peroxide-linked secondary antibodies, washed again in chemiluminescence (Chemicon) and exposed to Fuji Medical X-ray films. Lased-scanning densitometry (GelPro Analyzer Software, GraphPad) was used for the quantification of the films.

### 2.8. Midgut Bacterial Microbiota Analysis

Total DNA extraction was conducted in triplicate samples (~25 mg) per dietary treatment using the QIAGEN QIAmp DNA Mini Kit (Qiagen, Hilden, Germany), following the manufacturer's protocol. Bacterial DNA was amplified targeting the V3–V4 regions of the bacterial 16S rRNA gene. A MiSeq Illumina instrument (MRDNA Ltd., Shallowater, city, TX, USA, sequencing facilities) was used for the sequencing of the amplified sequences. The process of raw 16S rRNA sequences was performed according to the MOTHUR standard operating procedure (v.1.45.3), while the classification of the operational taxonomic units (OTUs) was conducted using the SILVA database.

### 2.9. Histological Examination of Liver and Gut

Immediately after euthanization, the liver of each fish was dissected, weighed, and fixed into Davidson solution at 4°C. The posterior and anterior gut segments were also dissected and fixed into the same solution. Dehydration of the samples was performed using a series of graded ethanol solutions, followed by immersion in xylol and then, embedding in paraffin. The sections that were taken were 5 μm in thickness, stained with hematoxylin–eosin, and examined under the microscope (Bresser Science TRM 301, Bresser GmbH, Rhede, Germany). Histological abnormalities were recorded and digital images were taken (Bresser MikroCam 5.0 MP, Bresser GmbH, Rhede, Germany).

### 2.10. Statistical Analysis

Values are presented as the mean ± standard error of the mean (S.E.M). Shapiro–Wilk's normality test and Levene's homogeneity test ware performed on growth, nutrient indexes, and biochemical parameters data. The statistical analysis was an analysis of variance (ANOVA) to test the null hypothesis that all experimental treatments (or diets) were sampled from populations of identical means. In case of rejection of the null hypothesis a Tukey's multiple comparison test was performed to find out differences between experimental treatments (or diets). Pearson correlation coefficients were calculated between dietary and tissue amino acid composition. The abovementioned statistical analyses were carried out using SPSS Statistics, version 26 (SPSS, Chicago, IL, USA). Furthermore, to determine any significant differences regarding fish gut microbiome, PERMANOVA (permutational multivariate ANOVA) was performed using PAST4.03 software. Significance was assigned at the 5% level.

## 3. Results

### 3.1. Growth Performance and Feed Utilization

There was a significant effect of diet on fish performance. Fish fed with the control and 0%FMFO were not different (*p*  > 0.05) and showed the highest WG, SGR, FCR, PER, and LER compared to the other diets. Fish fed with the IM diet had significantly lower final weight, WG, SGR and VFI values, but not different (*p* > 0.05) FCR, PER, and LER compared with the control and 0%FMFO treatments. Fish fed with the TM diet displayed the lowest growth, FI (g/fish), PER, LER, and higher FCR (*p* < 0.05). Hepatosomatic index and survival were not affected by any dietary treatment (*p* > 0.05; Table 2).

### 3.2. Fillet Proximate Composition, Amino Acid, and Fatty Acid Profiles

At the end of the experiment, diet showed a significant effect on the moisture content of white muscle (*p* < 0.05; Table 2). Moisture in white muscle was at lowest level in fish fed the IM diet and the highest level in fish fed with the TM diet, compared with those in the other dietary treatments. Protein content in the white muscle was not affected (*p*  > 0.05) by dietary treatment. There were no differences (*p* > 0.05) in lipid and energy levels in the fillets of the fish fed the control and 0%FMFO diets. On the other hand, the lipid and energy contents of the fish fed the IM diet were greater (*p* < 0.05) and those of the fish fed the TM diet were significantly lower than those of the other dietary treatments. In the TM treatment, the ash content significantly increased (*p*  < 0.05) compared to the control treatment, which presented the lowest values.

Muscle amino acid composition of seabream fed the experimental diets is shown in Table 2. Arginine, leucine, and phenylalanine decreased in the three experimental diets, compared to the fish fed the control diet. Histidine, isoleucine, and threonine increased in muscle of fish fed the 0%FMFO diet. The highest lysine value was detected in fish fed the TM diet, although these fish had the lowest levels of methionine, valine, and tyrosine, compared to the other dietary treatments. Regardless of the diet, arginine, leucine, lysine, and valine in the white muscle seem to be increased compared to the amino acid content of the respective administered diets. In contrast, histidine, methionine, threonine, and tyrosine in the muscle showed a decrease trend compared to those of the respective administered diets. Isoleucine detected in muscle of fish fed control, 0%FMFO, and IM diets was at higher level than those detected in each diet respectively. However, the isoleucine detected in fish fed TM diet displayed similar level to that of TM diet. Moreover, phenylalanine in the muscle of fish of the control dietary treatment was increased compared to the phenylalanine in the diet. In contrast, phenylalanine in 0%FMFO, IM, and TM dietary treatments decreased compared to the respective diets.

Pearson correlation indicated a positive correlation between amino acids of the experimental diets and the white muscle tissue (*r* = 0.712, *p*=0.002 for the control treatment; *r* = 0.739, *p*=0.001 for 0%FMFO treatment; *r* = 0.651, *p*=0.006 for IM treatment; *r* = 0.646, *p*=0.007 for TM treatment). In addition, the Pearson correlation coefficient indicated close relationships regarding the amino acids in the muscle of fish fed the control dietary treatment and those fed the 0%FMFO dietary treatment (*r* = 0.98, *p*=0.001), fed IM dietary treatment (*r* = 0.99, *p*=0.001), and fed TM dietary treatment (*r* = 0.992, *p*=0.001).

Fatty acids profile of white muscle is shown in Table 2. Dietary treatments did not have an effect on fatty acids concentration of 14:1, 16:1*n*−9, 16:1*n*−7, 18:0, 18:1*n*−7, 20:2*n*−9, 20:2*n*−6, 20:3*n*−6, 20:4*n*−6, and 20:4*n*−3 (*p* > 0.05). 14:0 and 16:0 decreased (*p* < 0.05) and 15:0 and 18:1*n*−9 increased in fish fed 0%FMFO compared to the rest dietary treatments. Fatty acids, 17:0, 18:2*n*−6, 18:3*n*−3, 20:0, 20:3*n*−3, and 22:6*n*−3 (DHA) increased (*p* < 0.05) in fish fed the diet without FM and FO (0%FMFO). These fish showed a significant decrease (*p* < 0.05) in 20:1*n*−9 and 22:1*n*−9. Furthermore, 17:1*n*−7, 20:5*n*−3 (EPA), 22:1*n*−7, and 24:1*n*−9 fatty acids were higher in fish fed IM diet, TM diet, and control diet. TM diet led to an increase (*p* < 0.05) of 18:2*n*−6 and 18:3*n*−6 fatty acids in fish muscle. Fish fed TM and 0%FMFO dietary treatments had decreased 18:4*n*−3 fatty acid in the muscle, compared to control and IM dietary treatments.

### 3.3. Enzymes of Intermediate Metabolism

The enzymatic activities of HOAD in the liver of seabream did not show significant differences (*p* > 0.05) among the dietary treatments (Table 3). However, HOAD activity in foregut increased in fish fed the 0%FMFO diet and decreased in fish fed the TM diet (Table 3). In the foregut, all experimental diets resulted to an increase in the L-LDH activity, compared to the control treatment, with the TM diet exhibiting the most increased values. Also, L-LDH activity in liver was significantly increased in fish fed the aforementioned diet. It should be underlined though that in the foregut, all experimental diets resulted to an increase in the HOAD activity, compared to the control treatment, with the TM diet exhibiting the most increased values. On the other hand, CS activity increased (*p* < 0.05) in the liver of all experimental dietary treatments compared to control, while in the foregut a significant decrease (*p* < 0.05) was observed in 0%FMFO treatment. No significant variations were observed in the IM and TM dietary treatments compared to the control.

### 3.4. Heat Shock Response, p38 MAPK, Akt, and AMPK Activation in the Liver

**Figure 1:**
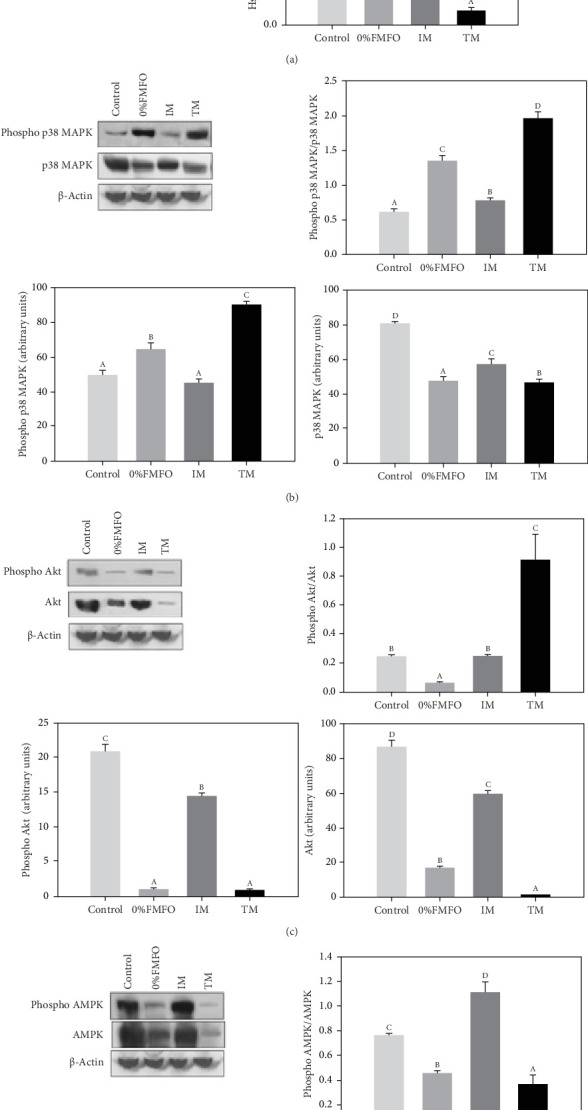
(a) Hsp70/β-actin and Hsp90/β-actin levels, (b) levels of phospho p38 MAPK and phospho p38 MAPK/p38 MAPK ratio, (c) levels of phospho Akt and Akt and phospho Akt/Akt ratio, and (d) levels of phospho AMPK and AMPK and phospho AMPK/AMPK rario in the liver of *Sparus aurata* under the effect of control, 0%FMFO, IM, and TM experimental diets. Representative blots are shown. Values are presented as means ± S.D. Lower case letters depict significant differences (*p* < 0.05) between the experimental diets.

There was a significant effect of the diet on the response of Hsp70 and Hsp90. Both levels decreased in all dietary treatments compared to control (Figure 1a). Specifically, the most significant decrease (*p* < 0.05) for both examined heat shock proteins (Hsps) was observed in the TM treatment, while the least significant decrease (*p* < 0.05) was observed in the IM dietary treatment. All dietary treatments exhibited statistically significant differences (*p* < 0.05) between all dietary regimes.

Regarding the p38 MAPK pathway, a contrary pattern was observed for the phospho p38 MAPK and p38 MAPK under the effect of the experimental dietary treatments (Figure 1b). Phospho p38 MAPK levels significantly increased (*p* < 0.05) under the effect of 0%FMFO and TM treatments compared to the control, while p38 MAPK levels significantly decreased (*p* < 0.05) in all dietary treatments. However, p38 MAPK phosphorylation ratio (phospho p38 MAPK/p38 MAPK) exhibited a different pattern. Levels significantly increasing (*p* < 0.05) in all experimental trials compared to control. Specifically, the most significant increase was observed in TM and the least significant increase in IM dietary group. Regarding p38 MAPK phosphorylation ratio, all dietary groups exhibited statistically significant differences (*p* < 0.05) between all dietary treatments.

Compared to the abovementioned pathways, a different pattern regarding Akt was apparent in the liver of *S*. *aurata* in response to the experimental dietary treatments. Specifically, both phospho Akt and Akt levels significantly decreased (*p* < 0.05) under the effect of all dietary treatments with the most intense observed in 0%FMFO and TM (Figure 1c). However, Akt phosphorylation ratio (phospho Akt/Akt) exhibited statistically significant increase (*p* < 0.05) only under the TM treatment, while a significant (*p* < 0.05) decrease was apparent in the 0%FMFO group compared to the control. IM dietary treatment resulted in no changes compared to the control group.

Regarding AMPK, both phospho and total levels exhibited a pattern of significant decrease (*p* < 0.05) under the effect of 0%FMFO and TM treatments compared to the control. Similarly, AMPK phosphorylation ratio (phosphor AMPK/AMPK) decreased (*p* < 0.05) in 0%FMFO and TM dietary treatments, while the IM dietary treatment resulted in a significant increase (*p* < 0.05) compared to the control (Figure 1d).

### 3.5. Midgut Bacterial Microbial Analysis

**Figure 2:**
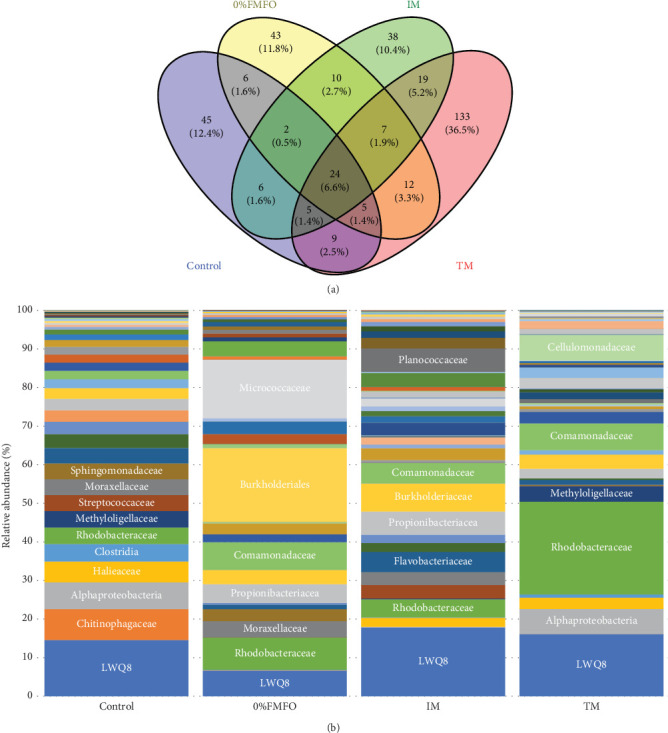
(a) Venn diagram of the unique and shared operational taxonomic units (OTUs) in the midgut of *Sparus aurata* under the effect of control, 0%FMFO, IM, and TM experimental diets and (b) relative abundance of bacterial families in the midgut of *S*. *aurata* fed the experimental diets.

Following the raw data processing, the average number of OTUs detected in the midgut samples of *S*. *aurata* fed the control, 0%FMFO, IM, and TM diets was not statistically significant (*p* > 0.05; Figure 2a). The number of unique OTUs was 45 in control dietary treatment, 45 in 0%FMFO, 38 in IM, and 133 in TM. Several bacterial families were identified in the midgut samples with relative abundance displaying variations among the different dietary treatments (Figure 2b). Chitinophagaceae, Alphaproteobacteria, Haliaceae, Clostridia, Methyloligellaceae, Streptococcaceae, Moraxellaceae, Splingomonadaceae, Flavobacteriaceae, Neisseriaceae, and Corynebacteriaceae were among the dominant bacterial families observed in midgut of fish fed control diet, while Burkholderiales, Micrococcaceae, Comamonadaceae, Propionibacteriaceae, Moraxellaceae, Bacteroidota, and Burkholderiaceae were among dominant families of fish fed 0%FMFO diet. In fish fed IM diet the midgut bacterial dominance consisted of Burkholderiaceae, Planococcaceae, Propionibacteraceae, Comamonadaceae, Flavobacteriaceae, Campylobacteraceae, Streptococcaceae, Moraxellaceae, Staphylococcaceae, and Xanthobacteraceae and in fish fed TM diet consisted of Comamonadaceae, Cellulomonadaceae, Alphaproteobacteria, Methyloligellaceae, Burkholderiaceae, and Vibrionaceae. Bacterial families LWQ8 and Rhodobacteraceae were detected among the dominant bacteria in midgut of fish of all dietary treatments.

The presence of Cellulomonadaceae was not observed in fish fed the 0%FMFO and IM diets, there was absence of Chitinophagaceae in fish fed IM diet, while Rubritaleaceae family was present only in fish of TM dietary treatment. The midgut bacteria richness increased in fish fed with the TM diet. Shannon and coverage indices also increased in fish of the TM dietary treatment, although evenness was reduced, compared to the other dietary treatments, but without statistical significance (*p* > 0.05).

### 3.6. Histological Examination

**Figure 3:**
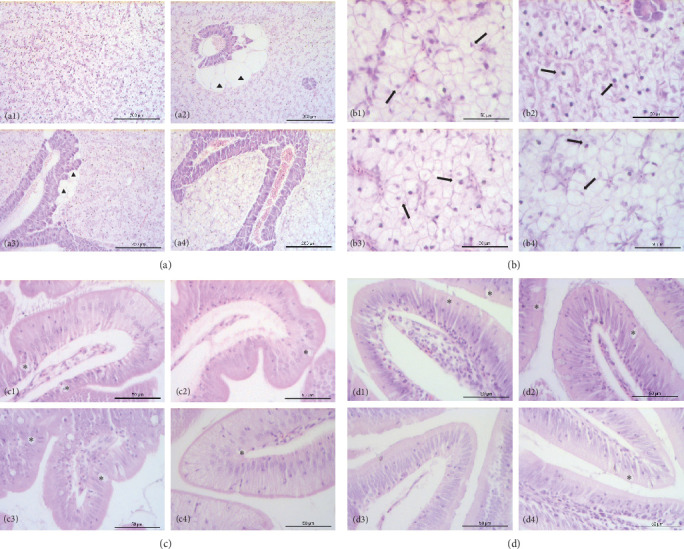
(a, b) Liver, (c) anterior gut, and (d) posterior gut histological examination of *Sparus aurata* fed the experimental diets (1) control, (2) 0%FMFO, (3) IM, and (4) TM, respectively. Fish fed experimental diets 0%FMFO (2) and IM (3) displayed large lipid droplets around pancreatic islets (▲). In addition, in fish fed the control (1) and experimental diets (2–4) was detected nuclei displacement (arrows) in liver. Anterior and posterior gut in fish in all dietary treatments had normal structure with goblet cells present (*⁣*^*∗*^).

The liver of fish fed the 0%FMFO and IM dietary treatments had large lipid droplets around the pancreatic islets (Figure 3a). Fish fed with the replacement diets as well as the control diet, showed mild lipid droplets accumulation and nuclei displacement detected in some cases compared to a normal liver imaging. Steatosis or hemorrhages were not detected (Figure 3b). Diet did not have a significant effect on anterior and posterior gut histology. All dietary treatments displayed a normal structure with normal distribution of goblet cells with no signs of inflammation (Figure 3c,d).

## 4. Discussion

In the current experiment, we showed that all dietary FM and FO can be replaced with a mixture of microalgae (*S. limacinum* and *P. tricornutum*), insect (*H. illucens*), and tunicate (*C. intestinalis*) without impairing growth performance or fish utilization in *S*. *aurata*. Nevertheless, fish growth and feed utilization were significantly suppressed when either *H. illucens* or *C*. *intestinalis* meal was added at 211 g/kg, replacing 68% and 46% of the dietary FM, respectively. These results indicate that high level of FM replacement by either IM or TM is not nutritionally adequate, and that the use of microalgal biomass is beneficial for gilthead sea bream performance. *Hermetia illucens* protein has been proven to be effective at replacing moderate levels of FM protein in the diet of this species. Replacing 30% of FM with a diet of 195 g/kg [25] did not impair gilthead sea bream growth or feed efficiency. Karapanagiotidis et al. [45] tested the use of a defatted instead of a full-fat *H. illucens* meal together with an inclusion level as high as 174 g/kg and a replacement of 30% of FM protein and found a decreased FI, which in turn impaired growth and feed efficiency, which is similar to the findings by Fabrikov et al. [43] for included 109–180 g/kg. Carvalho et al. [44] also observed reduced FI and growth performance in *S*. *aurata* fed diets with 10% *H*. *illucens*, replacing 66% of FM, suggesting that *S*. *aurata* may have a low ability to digest the IM at increased inclusion levels in the feed. In other studies, total FM replacement using as much as 450 g/kg *H. illucens* meal did not impair gilthead sea bream growth or feed efficiency [81], while Pulido-Rodriguez et al. [42] reported that even greater growth and feed efficiency were achieved when *H. illucens* was added at 324 g/kg diet. The high digestibility of *H. illucens* meal in gilthead sea bream [81, 82] may explain these effects. Chitin found in IM has been suggested as an antinutritional factor affecting the digestibility of nutrients. Though, in the present study the 0%FMFO diet and the IM diet had similar inclusion levels of the same IM *H*. *illucens* (20.75% inclusion in 0%FMFO and 21.14% inclusion in IM) their growth performance was different. Fish fed the 0%FMFO diet showed better SGR similar to the control diet than fish fed the IM diet. Therefore, chitin in the dietary treatment group fed 0%FMFO did not slow down or inhibit fish growth performance. Moreover, chitin in IM can have a functional role [29, 83].

Karapanagiotidis et al. [45] suggested that full fat black soldier fly meal at high inclusion levels in *S*. *aurata* diets may have negative effects on diet palatability demonstrated by reduced feed consumption and decreased nutrient intake due to incomplete catabolism in fish. Defatting process of *H*. *illucens* meal can affect the availability of lipids, becoming bound in the chitin structure [69], especially the MUFA. Pulido-Rodriguez et al. [42] examined the use of partially defatted black soldier fly prepupae meal in *S*. *aurata* appetite-related genes and found that the *H*. *illucens* meal, even at the inclusion level of 324 g/Kg did not depress the central neuroendocrine mechanism involved in appetite stimulus, resulting in FI, SGR, and FCR similar to the control treatment. According to Randazzo et al. [70], partially defatted black soldier fly prepupae meal increased glycosylated compounds in the midgut and lipid deposition in liver and of *S*. *aurata*, with the latter associated with the fatty acid profile of the IM. Furthermore, the *H*. *illucens* meal has a balanced EAA profile [34, 68] and contains bio-active compounds, like medium-chain fatty acids and chitin [29, 83–85], which can stimulate the immune system of fish, providing antimicrobial and anti-inflammatory properties [28, 36].

The addition of *P*. *tricornutum* in combination to *Schizochytrium* sp., IM and TM to the diet of gilthead sea bream had a positive effect on the growth performance of the fish. When *P*. *tricornutum* meal replaced FM in starter diets for *S. aurata* at 4% level, fish SGR was unaffected but an evident negative effect on survival was observed [8]. On the other hand, Ribeiro et al. [86] reported that in finishing diets of gilthead sea bream, the inclusion of 2.5% *P. tricornutum* did not impair growth or feed utilization parameters, improved fish skin pigmentation or acted as a functional ingredient in the diet. Reis et al. [87] examined the use of *P*. *tricornutum* meal, either in terms of whole cell or broken cell biomass, at the 1% level in a FM-free diet for juvenile gilthead sea bream and found that growth performance parameters and feed efficiency remained unaffected, while the immune system of fish was stimulated, improving fish health, especially in the case of broken cell biomass. The low level of inclusion of *P*. *tricornutum* in the diet of gilthead sea bream at different stages of development seems to be beneficial to the fish. In the present study, the use of 8% *Schizochytrium* sp. blended with other ingredients in the diet of gilthead sea bream did not affect the growth performance of the fish. Ganuza et al. [14] reported that a lower inclusion level (2.5%) of *Schizochytrium* sp. in microdiets for *S. aurata*, as a DHA source, did not affect the larvae performance; although fish growth and survival were reduced when *Schizochytrium* sp. replaced FO fully in the diet. In contrast, Carvalho et al. [88] reported that the total replacement of FO by commercial products based on *Schizochytrium* sp. in weaning microdiets for *S*. *aurata* improved growth performance and had no effect on survival, but increased the survival rate under stressful conditions, probably due to the increased DHA content of *Schizochytrium* sp. Similar results on the survival rate were reported by Eryalçin and Yildiz [89], who replaced 10% of FM and all FO with a mixture of microalgae containing *P*. *tricornutum*, *Schizochytrium* sp., *Tetraselmis suecuca*, *Isochrysis* sp., and *Nannochloropsis oculata* meal, in weaning microdiets of gilthead sea bream, allowing growth performance to be unaffected. According to Santigosa et al. [15], the growth performance of seabream with an initial weight of 64.5 g fed 3.5% *Schizochytrium* sp. oil as a replacement for FO was also unaffected, indicating that the supplementation of feed with microalgae could lead to a diet rich in *n*−3 and *n*−6 fatty acids and environmentally friendly. A mixture of *Schizochytrium* sp. with other microalgae, such as *Microchloropsis gaditana*, has also been shown to be effective for total FO replacement in diet for juvenile gilthead sea bream without influencing growth and feed utilization, providing valuable EPA and DHA to the fish [19]. Improved growth performance and feed utilization were seen in gilthead sea bream when fed a diet containing a blend of microalgae (3.2% *Schizochytrium* sp., 5% *Chlorella* sp., and 0.5% *Tetraselmis* sp.), 0.4% macroalgae, and 0.02% selenized yeast replacing 37% of FM. Nevertheless, when the dietary levels of microalgae and yeast were doubled, this led to impaired growth [90].

In the present study, in order to formulate the 0%FMFO diet that was devoid of FM and FO, the levels of low protein binder meals, such as horsebean meal and wheat meal were also reduced, as the novel ingredients' protein level is lower that of FM present in the control diet. The variation in the dietary horsebean meal is not expected to have contributed to the variation in fish performance as it has been shown up to 35% inclusion of horsebeans in the diet did not compromise the growth performance of *S*. *aurata* [91] or the digestibility of dietary protein and lipids in *D*. *labrax* [92, 93].

There is limited research on the replacement of FM with TM in the diet of gilthead sea bream. In the present study, *S*. *aurata* fed a TM diet had increased FCR and decreased SGR. Similar results were also shown in *Salmo salar* fed TM at 200 g/kg, which was 45% replacement for FM and this increase in FCR occurred due to the high ash content of TM [46]. The present results indicate that TM in the diet of seabream in the present study decreased the fish FI and LER, thus, decreased nutrient and energy intake, resulted in decreased lipid content in fish fillets, while IM increased the lipid content in fish muscle. Increased lipid levels in muscle of fish fed IM diet cannot be related to increased FI, because the FI in this treatment decreased, compared to control, indicating that dietary lipid derived from IM was not catabolized, but deposit in high degree in fish muscle. Kousoulaki et al. [46] also observed increased lipid content in muscle of *S*. *salar* fed 20% *H*. *illucens* meal diet, as a result of increased amino acid catabolism for energy use. However, Fabrikov et al. [43] reported that 50% replacement of FM with *H. illucens* meal led to decreased lipid content in *S*. *aurata* fillets. The latter may be attributed to differences in feed digestibility and the level of dietary FM replacement. In the present study, the amino acid deposition in the muscle of *S*. *aurata* remained unaffected by the inclusion of algae meal in combination to IM and TM in diets. The accumulation of DHA in the fish fillets increased in the fish fed the IM diet and was similar to that in the control diet-fed fish. These results agree with those of Mastoraki et al. [25] and Pulido et al. [94], who reported that the inclusion of *H. illucens* in *S*. *aurata* and *D*. *labrax* in the diets did not affect the content of main fatty acids. In addition, Carvalho et al. [44] reported that low-level inclusion of *H. illucens* did not affect the proximate composition, amino acid, or fatty acid profile of *S*. *aurata*. Atalah et al. [8] observed an increase in the lipid content of *S. aurata* when they were fed weaning diets containing *P*. *tricornutum* meal as a replacement for FM. Furthermore, gilthead sea bream fed a mixture of macroalgae, microalgae containing 3.2% *Schizochytrium* sp. and selenized yeast to replace 33% of the FM had a positive effect on *n*−3 PUFA accumulation in fish muscle, leading to an increase in the fatty acid content of the fillet [90]. Similar results were also found in gilthead sea bream fillets, either fed 3.5% *Schizochytrium* sp. oil [15] or a blend containing *Schizochytrium* sp. and *P. tricornutum* meals [89]. The successful FO substitution with *Schizochytrium* sp. meals in salmon feeds demonstrates the feasibility of low trophic level organism to promote the sustainability of the aquaculture sector [95].

Differences in the activity levels of enzymes involved in intermediary metabolism in the liver and foregut of fish fed the experimental diets indicated alterations in the metabolic pathways due to dietary ingredients. Increased L-LDH activity in fish fed TM indicates hepatic and intestinal lactate uptake, which may suggest a glycolytic carbohydrate metabolism dependence [60]. The 0%FMFO diet containing microalgae meal had no effect on hepatic HOAD activity, which is in agreement with the HOAD activity levels in the liver of gilthead sea bream fed nutraceuticals derived from microalgae [96]. On the contrary, HOAD activity levels in the present study increased in the foregut, indicating lipid oxidation. Fish fed the IM diet exhibited increased L-LDH and CS activity levels in the foregut, contrary to decreased CS and L-LDH activities in the foregut of gilthead sea bream fed the *Tenebrio molitor* diet [97]. According to Antonopoulou et al. [80], the increase in HOAD activity in relation to unaffected levels of L-LDH and CS indicate that liver metabolism mainly utilizes lipid energy reserves.

Hsp70 and Hsp90 levels in the IM, TM, and 0%FMFO diets significantly decreased compared to the control, contrary to the upregulated Hsp70 in *Cyprinus carpio* fed diets with 75%–100% FM replacement with defatted *H. illucens* [63]. Similarly, *Oncorhynchus mykiss* fed a 50% full-fat *H. illucens* diet exhibited increased Hsp70 levels in the liver, suggesting a possible stress response [97]. The decreased Hsp levels in the present study in the liver of fish fed IM, TM, and 0%FMFO diets could be attributed to the fact that only significant changes in the quantity of amino acids provided by food is necessary for an alteration in Hsp levels [60], whereas amino acid deprivation can lead to Hsp deficiency [98]. However, this needs to be further investigated. Although the involvement of p38 MAPK in the induction of Hsp70 has been proven in fish tissues [99, 100] contradictory results were obtained in the present study, with p38 MAPK being activated in the liver of all experimental diet groups. p38 MAPK phosphorylation likely occurs when dietary lipids cannot meet fish dietary requirements, thus, indicating a stress response [80]. Akt activation, possessing a key role in glucose metabolism [101], increased in the TM compared to the control group, and along with the increased L-LDH activity levels confirmed in TM induced carbohydrate metabolism. Similarly, Liu et al. [102] reported that upregulated hepatic Akt is a potential cause of elevated amino acid metabolism in *O*. *mykiss* fed a mixture of IM (*Hermetia illucens*) and *Chlorella sorokiniana*. Contrary to Akt phosphorylation, AMPK activation was more apparent in the IM dietary group, as also evidenced by HOAD, probably due to increased lipid exploitation processes [103].

With respect to the gut microbiota, there was no statistical difference of midgut bacterial taxa that detected in fish fed the 0%FMFO, IM, or TM diet, which contained *H. illucens*, *C*. *intestinalis*, *P*. *tricornutum*, and *S*. *limacinum* meal (separately or mixed), compared to the control diet. In contrast, Cerezuela et al. [9] reported decreased bacterial activity in the intestine of *S*. *aurata* fed *P*. *tricornutum* meal, suggesting a fragile gut microbial community. However, Piazzon et al. [104] observed a strong interaction between the gut microbiome and *S*. *aurata* fed a diet with IM and algae oil, promoting growth and health. Regarding the bacterial richness in *S*. *aurata* fed a diet with *H. illucens* meal, no differences were detected, although changes in the metabolic pathways of the bacterial community may occur [40], even at 50% replacement of FM [43]. According to Katsoulis-Dimitriou et al. [20], the gut microbiota of *S*. *aurata* fed diets containing microalgae, were able to adapt to these diets and metabolized fucose, which is a major carbohydrate that can be found in microalgae cells. This ability of gut microbiota to utilize the microalgae fucose has potentially beneficial results to the host, by inducing the short chain fatty acids production [105–107].

The gut microbiota potentially plays an important role in the nutrition of fish by enzyme production [108]. In the present study, the observed dominant bacterial families in fish midgut may have nutritional role, producing enzymes (Bacillaceae [109], Flavobacteriaceae [110, 111], Rhodobacteraceae [112], Cellulomonadaceae [113], and Saccharimonadales in which the LWQ8 family belongs [114]), short-chain fatty acids (Clostridia [115], Halieaceae [116], and Propionibacterium [117]), amino acids (Halieaceae [116], Planococcaceae [118], and Neisseriaceae [119]), vitamins (Propionibacterium [117] and Neisseriaceae [119]), and assist to metabolism (Micrococcaceae [120], Moraxellaceae [121], and Rubritaleaceae [122]) and growth (Micrococcaceae [123], Comamonadaceae [124], and Planococcaceae [125]). Other bacterial families are found to improve the immune system and health status (Streptococcaceae [126], Alphaproteobacteria [127], Burkholderiaceae [120], *Propionibacterium* [117], and Comamonadaceae [124]), while other are involved in nitrogen cycle (Sphingomonadaceae [128, 129]) and CH_4_ oxidation (Methyloligellaceae [130]). Chitinophagaceae bacteria known for their ability to degrade chitin [131] were absent in IM dietary group. This observation may be the result of microbial shifts derived from the rearing substrate, life stage, and processing of the insect, in combination with the microbial adaptation to the host metabolic requirements [34, 132, 133]. Also, Kroeckel et al. [69] did not observe any chitinolytic active bacteria in the midgut of *Psetta maxima* fed *H*. *illucens* meal diet. On the other hand, Flavobacteriaceae, which includes several members known to produce amylase, protease, and chitinase [110, 111], were observed d in fish fed IM. The ability to break down chitin favored its presence in the intestine of fish fed the IM diet, thus, providing valuable nutrients to the fish.

Lipid droplets were found around the pancreatic islets in the 0%FMFO and IM experimental diet groups, while nuclear displacement was observed in the liver of the examined fish. The presence of lipid droplets in pancreatic islets of fish fed IM diet, could correlated to increased lipid content observed in fish muscle of the abovementioned dietary group. Abundant lipid droplets were also found in *S*. *aurata* fed a *H. illucens* diet without altering lipid accumulation [70]. In contrast, the inclusion of *H. illucens* meal in the *S*. *salar* diet did not affect lipid droplets in the liver [38]. Additionally, Karapanagiotidis et al. [19] reported no alterations in the hepatic histology of seabream fed *Schizochytrium* sp. or *Microchloropsis gaditana* as a FO replacement. Instead, Di Rosa et al. [41] reported severe alterations in the posterior gut structure of *S*. *aurata* fed 50% *H. illucens* meal, which may be due to increased levels of chitin in the diet.

## 5. Conclusion

Total replacement of FM and FO with a blend of IM, microalgae, *Schizochytrium* sp., and TM did not affect gilthead sea bream growth performance compared with the control diet, although replacement of 68% of the FM with IM and replacement of 45% of the FM with TM alone resulted in reduced fish growth performance. The CS activity in the liver increased in the fish of the aforementioned treatments, indicating the use of the Krebs cycle for energy production. In addition, the livers of fish fed the TM diet exhibited an increase in L-LDH and a decrease in Hsps, AMPK, and p38 MAPK activity, implying a reduced stress response. Bacterial taxa present in the midgut of fish from all experimental groups, were involved in fish metabolism and nutrient supply to the host. The differences in fish metabolism and the gut microbiota seemed to be linked to the differences in the experimental diets. According to the histological examination, large lipid droplets appeared around the pancreatic islets of the fish fed the 0%FMFO and IM diets, although mild lipid droplet accumulation was detected in the liver of the fish fed all the experimental diets (replacement diets and the control diet). To conclude, new horizons are opened for the replacement of FM and FO in *S*. *aurata* feeds by incorporating into the aquafeed a mixture of innovative ingredients that can be grown sustainably on agricultural sidestream biomasses.

## Figures and Tables

**Table 1:** Formulation, proximate composition (%), amino acid ratio (A/E) profile, and fatty acids (%) profile of the experimental diets Control, 0%FMFO, IM and TM.^a,b^

Ingredients (%)	Control	0%FMFO	IM	TM
Formulation				
Fish meal organic^c^	26.55	0	8.47	14.36
Krill hydrolysate	0	2.07	2.11	2.11
* Schizochytrium limacinum* ^d^	0	7.99	0	0
* Phaeodactylum tricornutum* ^e^	0	7.78	0	0
* Hermetia illucens* ^f^	0	20.75	21.14	0
* Ciona intestinalis* ^g^	0	20.75	0	21.12
Wheat gluten	17.97	15.32	17.45	19.53
Wheat	17.1	5.19	12.05	5.28
Horsebeans	16.99	2.07	16.91	14.79
Rapeseed lecithin	1.06	1.35	0.9	1.8
Fish oil organic^h^	10.42	0	12.1	9.47
Rapeseed oil	1.02	4.57	0	1.19
Linseed oil	0.15	1.85	0	0.9
Histidine	0.82	0.62	0.59	0.81
L-Lys 79%	0.71	0.76	0.72	0.74
L-Threonine	0.41	0.21	0.4	0.31
DL-Methionine	0.25	0.35	0.37	0.31
Choline chloride	0.53	0.52	0.53	0.53
Vitamin premix^i^	2.65	2.59	2.64	2.64
Mineral premix^j^	2.93	2.40	2.71	3.13
Vitamin C	0.05	0.05	0.05	0.05
Cholesterol	0.53	0.52	0.53	0.53
Water	−0.14	2.30	0.35	0.41
Proximate composition in DM				
Moisture	7.5	6.5	9.5	7.7
Proteins	46.4	43.6	43.7	43.3
Lipids	15.4	15.0	15.2	14.3
Ash	6.7	11.5	5.9	11.1
Energy (Kj/g)	22.6	21.6	23.2	21.7
Amino acid ratio (A/E) profile				
EAA ratio (% of total EAA)^k^				
Arginine	12.03	10.79	11.26	11.39
Histidine	7.59	7.27	5.29	6.20
Isoleucine	8.26	8.74	8.61	9.08
Leucine	15.95	14.93	15.64	16.40
Lysine	14.15	12.55	13.53	13.21
Methionine	5.37	4.96	5.90	5.18
Phenylalanine	9.76	10.28	10.01	10.26
Threonine	10.07	9.71	9.80	10.20
Valine	9.91	11.01	10.61	10.52
Tyrosine	6.92	9.76	9.36	7.57
NEAA ratio (% of total NEAA)^l^				
Alanine	10.35	9.66	9.87	9.16
Aspartic acid	13.82	15.76	14.23	13.94
Glycine	9.06	9.56	9.11	8.76
Serine	9.02	9.20	9.44	9.41
Glutamic acid	44.16	40.32	42.36	44.34
Hydroxyproline	0.89	1.02	0.95	1.01
Proline	14.30	14.74	14.90	14.54
Cystein + cystine	2.66	3.16	2.69	3.43
Ornithine	0.22	0.25	0.24	0.25
EAA/NEAA	0.86	0.89	0.87	0.80
Fatty acids (% of total)				
12:0	—	1.7	1.53	—
14:0	4.96	2.41	5.77	5.32
14:01	0.04	—	0.05	0.04
15:0	0.35	0.82	0.35	0.42
16:0	13.61	23.21	14.7	14.87
16:1*n*−7	3.39	2.54	4.06	3.52
16:1*n*−9	0.14	0.06	0.2	0.14
17:0	0.18	0.41	0.19	0.24
17:1*n*−7	0.2	—	0.2	—
18:0	1.67	2.32	1.67	2.31
18:1*n*−7	1.92	2.09	1.65	2.20
18:1*n*−9	17.42	28.03	14.65	21.35
18:2*n*−6	10.08	15.68	10.52	6.77
18:3*n*−3	2.21	9.23	1.63	1.56
18:3*n*−6	0.05	—	0.06	—
18:4*n*−3	1.84	0.18	1.97	0.41
20:0	0.26	0.44	0.23	0.38
20:1*n*−9	10.56	1.13	10.58	10.54
20:1*n*−11	0.91	0.13	1.05	1.24
20:2*n*−6	0.15	—	0.14	—
20:3*n*−3	0.05	—	—	—
20:4*n*−6	0.23	0.36	0.21	—
20:5*n*−3	4.14	1.25	4.12	3.76
22:0	0.14	0.22	0.13	0.25
22:1*n*−7	1.42	0.16	1.22	1.62
22:1*n*−9	17.2	0.93	17.05	17.36
22:5*n*−3	0.38	—	0.37	—
22:6*n*−3	5.43	6.48	4.78	4.93
24:1*n*−9	0.99	0.18	0.81	0.92
Total *n*−3	14.07	17.16	12.87	10.67
Total *n*−6	10.52	16.05	10.93	6.77
*n*−3/*n*−6	1.34	1.07	1.18	1.58

^a^More details on the proximate composition, amino acid, fatty acid profile, et cetera, of the algae meal, *Schizochytrium* sp. meal, insect meal, and tunicate meal, that were used in this study, someone can find in supplementation materials in Kousoulaki et al. [46].

^b^The amino acids histidine, L-lysine, L-threonine, and DL-methionine were added in all diets in order to meet *Sparus aurata* amino acid requirements.

^c^Pelagia (Egersund, Norway).

^d^Alltech Inc. (Dunboyne, Ireland).

^e^NORCE (Bergen, Norway).

^f^INNOVAFEED (Gouzeaucourt, France).

^g^Marine Feed AB (Stenungsund, Sweden).

^h^Måløy (Norway).

^i^Vitamin premix consisted of biotin, folic acid, niacin, pantothenic acid, pyridoxin, riboflavin, thiamin, vitamin B12, tocopherol acetate, and vitamin K.

^j^Mineral premix consisted of Se, Cu, Mn, and Zn in organic form.

^k^EAA of each essential amino acid = essential amino acid/sum of essential amino acids × 100.

^l^NEAA of each nonessential amino acid = nonessential amino acid/sum of nonessential amino acids × 100.

**Table 2:** Growth performance, nutrient utilization, and somatic indices, proximate composition (% of wet weight), essential amino acid ratio (% of total essential amino acids), and fatty acid profile of *Sparus aurata* white muscle under the effect of experimental diets.

	Control	0%FMFO	IM	TM
Growth performance, nutrient utilization, and somatic indices				
Final weight (g)	28.41^c^ ± 0.43	28.86^c^ ± 0.47	26.4^b^ ± 0.47	19.83^a^ ± 0.37
Weight gain (g)	21.87^c^ ± 0.43	22.19^c^ ± 0.47	19.84^b^ ± 0.47	13.30^a^ ± 0.37
Feed intake (g/fish)	17.95^b,c^ ± 0.42	19.36^c^ ± 0.75	16.56^b^ ± 0.43	13.96^a^ ± 0.53
Voluntary feed intake (VFI, %BW/day)	1.62^a,b^ ± 0.02	1.73^c^ ± 0.02	1.59^a^ ± 0.02	1.69^b,c^ ± 0.02
SGR (%/day)	2.25^c^ ± 0.02	2.21^c^ ± 0.02	2.09^b^ ± 0.02	1.65^a^ ± 0.03
FCR	0.89^a^ ± 0.02	0.95^a^ ± 0.02	0.93^a^ ± 0.3	1.51^b^ ± 0.23
PER	2.62^b^ ± 0.05	2.62^b^ ± 0.05	2.73^b^ ± 0.06	2.19^a^ ± 0.06
LER	7.85^b^ ± 0.15	7.59^b^ ± 0.15	7.84^b^ ± 0.18	6.64^a^ ± 0.18
Survival (%)	91.71^a^ ± 2.88	97.82^a^ ± 0.58	95.86^a^ ± 1.44	96.13^a^ ± 1.09
Hepatosomatic index	2.58^a^ ± 0.37	2.62^a^ ± 0.19	2.83^a^ ± 0.36	3.31^a^ ± 0.31
Proximate composition (% of wet weight)				
Moisture	72.81^b^ ± 0.4	72.34^a,b^ ± 0.4	71.1^a^ ± 0.44	74.76^c^ ± 0.43
Proteins	18.05^a^ ± 0.06	18.46^a^ ± 0.01	18.32^a^ ± 0.13	18.02^a^ ± 0.09
Lipids	6.87^b^ ± 0.04	6.74^b^ ± 0.18	8.47^c^ ±0.14	5.11^a^ ± 0.06
Energy (kJ/g)	7.09^b^ ± 0.00	7.10^b^ ± 0.02	7.68^c^ ± 0.01	6.27^a^ ± 0.00
Ash	1.24^a^ ± 0.05	1.43^a,b^ ± 0.04	1.40^a,b^ ± 0.04	1.50^b^ ± 0.06
Essential amino acid ratio (% of total essential amino acids)				
EAA ratio (% of total EAA)^1^				
Arginine	15.01	13.30	13.92	14.87
Histidine	1.88	2.70	2.56	2.67
Isoleucine	9.29	9.68	9.43	9.09
Leucine	18.19	17.93	17.89	17.92
Lysine	18.05	18.26	18.75	19.43
Methionine	0.33	0.33	0.34	0.29
Phenylalanine	9.84	9.72	9.56	9.35
Threonine	8.18	9.03	8.47	8.25
Valine	12.72	12.75	12.58	11.94
Tyrosine	6.51	6.29	6.51	6.19
NEAA ratio (% of total NEAA)^2^				
Alanine	8.89	9.56	9.29	8.57
Aspartic acid	22.23	25.67	21.60	24.48
Glycine	22.56	18.55	25.97	23.35
Serine	8.07	7.91	6.53	7.89
Glutamic acid	27.36	27.13	26.06	25.36
Proline	10.90	11.17	10.55	10.35
Fatty acids (% of total)				
12:0	—	1.57^b^ ± 0.04	0.69^a^ ± 0.24	0.27^a^ ± 0.01
14:0	4.12^b^ ±0.14	2.46^a^ ± 0.04	4.10^b^ ± 0.44	2.84^a,b^ ± 0.06
14:01	0.09^a^ ± 0.02	0.05^a^ ± 0.00	0.08^a^ ± 0.01	0.06^a^ ± 0.00
15:0	0.29^a^ ± 0.01	0.48^b^ ± 0.01	0.31^a^ ± 0.00	0.34^a^ ± 0.00
16:0	18.30^b^ ± 0.23	16.70^a^ ±0.20	18.37^b^ ± 0.29	17.70^a,b^ ±0.08
16:1*n*−7	5.39^a^ ± 0.26	4.47^a^ ± 0.09	5.28^a^ ± 0.03	4.51^a^ ± 0.06
16:1*n*−9	0.61^a^ ± 0.00	0.40^a^ ± 0.06	0.49^a^ ± 0.05	0.57^a^ ± 0.01
17:0	0.14^a^ ± 0.00	0.30^c^ ± 0.01	0.18^a,b^ ± 0.01	0.21^b^ ± 0.00
17:1*n*−7	0.22^a,b^ ± 0.00	0.17^a^ ± 0.01	0.23^b^ ± 0.01	0.21^a,b^ ± 0.00
18:0	3.05^a^ ± 0.30	3.38^a^ ± 0.01	3.14^a^ ± 0.13	3.39^a^ ± 0.05
18:1*n*−7	2.70^a^ ± 0.12	2.71^a^ ± 0.04	2.51^a^ ± 0.02	2.80^a^ ± 0.05
18:1*n*−9	24.06^a^ ± 0.08	30.69^b^ ± 0.34	23.87^a^ ± 1.37	27.99^a,b^ ± 0.48
18:2*n*−6	8.18^a^ ± 0.17	14.61^c^ ± 0.18	9.73^a,b^ ± 0.71	11.75^b^ ± 0.13
18:2*n*−9	0.44^a,b^ ± 0.05	0.26^a^ ± 0.07	0.30^a^ ± 0.07	0.78^b^ ± 0.02
18:3*n*−3	1.72^a^ ± 0.01	7.98^c^ ± 0.09	2.31^a^ ± 0.42	3.75^b^ ± 0.22
18:3*n*−6	0.25^a^ ± 0.03	0.29^a,b^ ± 0.04	0.22^a^ ± 0.04	0.51^b^ ± 0.03
18:4*n*−3	1.32^b^ ± 0.06	0.40^a^ ± 0.04	1.16^b^ ± 0.09	0.67^a^ ± 0.00
20:0	0.17^a^ ± 0.00	0.28^c^ ± 0.00	0.17^a^ ± 0.00	0.20^b^ ± 0.00
20:1*n*−9	8.32^c^ ± 0.23	1.38^a^ ± 0.01	7.27^b,c^ ± 0.64	6.03^b^ ± 0.13
20:2*n*−9	0.25^a^ ± 0.05	0.19^a^ ± 0.01	0.19^a^ ± 0.03	0.26^a^ ± 0.02
20:2*n*−6	0.21^a^ ± 0.00	0.23^a^ ± 0.03	0.23^a^ ± 0.03	0.20^a^ ± 0.00
20:3*n*−6	0.11^a^ ± 0.00	0.15^a^ ± 0.00	0.15^a^ ± 0.01	0.23^a^ ± 0.05
20:4*n*−6	0.27^a^ ± 0.03	0.45^a^ ± 0.01	0.32^a^ ± 0.05	0.35^a^ ± 0.03
20:3*n*−3	0.06^a^ ± 0.00	0.21^b^ ± 0.02	0.09^a^ ± 0.01	0.10^a^ ± 0.00
20:4*n*−3	0.51^a^ ± 0.00	0.31^a^ ± 0.01	0.55^a^ ± 0.10	0.32^a^ ± 0.01
20:5*n*−3	2.95^c^ ± 0.23	1.40^a^ ± 0.02	3.26^c^ ± 0.05	2.21^b^ ± 0.03
22:0	0.06 ± 0.00	0.16 ± 0.00	0.08 ± 0.00	0.08 ± 0.00
22:1*n*−7	1.31^b^ ± 0.08	0.30^a^ ± 0.02	1.07^b^ ±0.13	0.88^b^ ±0.07
22:1*n*−9	8.73^c^ ± 0.26	0.69^a^ ± 0.04	7.02^b,c^ ± 0.82	5.23^b^ ± 0.37
22:6*n*−3	5.3^a^ ± 0.37	6.86^b^ ± 0.12	5.96^a,b^ ± 0.33	4.97^a^ ± 0.07
24:0	—	0.07 ± 0.00	—	—
24:1*n*−9	0.8^b^ ± 0.04	0.35^a^ ± 0.00	0.62^b^ ± 0.03	0.58^a,b^ ± 0.07
Total *n*−3	11.88	17.17	13.34	12.02
Total *n*−6	9.03	15.74	10.66	13.05
*n*−3/*n*−6	1.31	1.09	1.25	0.92

*Note:* Values are presented as means and standard error of means. Means sharing the same superscript are not significantly different from each other (*p* > 0.05).

Abbreviations: FCR, feed conversion ratio; LER, lipid efficiency ratio; PER, protein efficiency ratio; SGR, specific growth rate.

^1^EAA of each essential amino acid = essential amino acid/sum of essential amino acids × 100.

^2^NEAA of each nonessential amino acid = nonessential amino acid/sum of nonessential amino acids × 100.

**Table 3:** Enzyme activities in liver of *Sparus aurata* fed with experimental diets and enzyme activities in foregut of *S*. *aurata* fed with experimental diets.

Enzyme activity (μmols of substrate min^−1^ g wet tissue^−1^)	Control	0%FMFO	IM	TM
HOAD	1.82^a^ ± 0.15	1.85^a^ ± 0.44	2.37^a^ ± 0.35	2.29^a^ ± 0.21
L-LDH	8.40^a^ ± 2.11	8.24^a^ ± 0.88	8.27^a^ ± 0.58	14.26^b^ ± 0.43
CS	0.68^a^ ± 0.10	2.72^b^ ± 0.36	2.12^b^ ± 0.10	2.36^b^ ± 0.29

**Enzyme activity (μmols of substrate min^−1^ g wet tissue^−1^)**	**Control**	**0%FMFO**	**IM**	**TM**

HOAD	0.91^a,b^ ± 0.009	3.32^c^ ± 0.34	1.43^b^ ± 0.08	0.19^a^ ± 0.01
L-LDH	37.63^a^ ± 0.98	40.14^a,b^ ± 1.53	44.61^b,c^ ± 0.51	47.57^c^ ± 1.36
CS	12.59^b^ ± 1.19	3.13^a^ ± 1.15	12.85^b^ ± 0.40	11.20^b^ ± 0.43

*Note:* Values are presented as means ± standard error of means. Means sharing the same superscript are not significantly different from each other (*p* > 0.05).

## Data Availability

The data that support the findings of this study are available from the corresponding author upon reasonable request.

## References

[1] Nagappan S., Das P., AbdulQuadir M. (2021). Potential of Microalgae as a Sustainable Feed Ingredient for Aquaculture. *Journal of Biotechnology*.

[2] Idenyi J. N., Eya J. C., Nwankwegu A. S., Nwoba E. G. (2022). Aquaculture Sustainability Through Alternative Dietary Ingredients: Microalgal Value-Added Products. *Engineering Microbiology*.

[3] Ansari F. A., Guldhe A., Gupta S. K., Rawat I., Bux F. (2021). Improving the Feasibility of Aquaculture Feed by Using Microalgae. *Environmental Science and Pollution Research*.

[4] Gilbert-López B., Barranco A., Herrero M., Cifuentes A., Ibáñez E. (2017). Development of New Green Processes for the Recovery of Bioactives From *Phaeodactylum tricornutum*. *Food Research International*.

[5] Sørensen M., Berge G. M., Reitan K. I., Ruyter B. (2016). Microalga *Phaeodactylum tricornutum* in Feed for Atlantic Salmon (*Salmo salar*) -Effect on Nutrient Digestibility, Growth and Utilization of Feed. *Aquaculture*.

[6] Barreto A., Pinto W., Rodrigues A. (2021). *Phaeodactylum tricornutum* Biomass in Microdiets Enhances Senegalese Sole (*Solea senegalensis*) Larval Growth Performance During Weaning. *Journal of Applied Phycology*.

[7] Sevgili H., Sezen S., Yılayaz A. (2019). Apparent Nutrient and Fatty Acid Digestibilities of Microbial Raw Materials for Rainbow Trout (*Oncorhynchus mykiss*) with Comparison to Conventional Ingredients. *Algal Research*.

[8] Atalah E., Cruz C. M. H., Izquierdo M. S. (2007). Two Microalgae *Crypthecodinium cohnii* and *Phaeodactylum tricornutum* as Alternative Source of Essential Fatty Acids in Starter Feeds for Seabream (*Sparus aurata*). *Aquaculture*.

[9] Cerezuela R., Fumanal M., Tapia-Paniagua S. T., Meseguer J., Morinigo M. Á., Esteban M. Á. (2012). Histological Alterations and Microbial Ecology of the Intestine in Gilthead Seabream (*Sparus aurata* L.) Fed Dietary Probiotics and Microalgae. *Cell and Tissue Research*.

[10] Stuart K. R., Barrows F. T., Silbernagel C., Alfrey K., Rotstein D., Drawbridge M. A. (2021). Complete Replacement of Fish Oil and Fish Meal in the Diet of Juvenile California Yellowtail *Seriola Dorsalis*. *Aquaculture Research*.

[11] Samuelsen T. A., Oterhals Å., Kousoulaki K. (2018). High Lipid Microalgae (*Schizochytrium* sp.) Inclusion as a Sustainable Source of n-3 Long-Chain PUFA in Fish Feed—Effects on the Extrusion Process and Physical Pellet Quality. *Animal Feed Science and Technology*.

[12] Annamalai S. N., Das P., Thaher M. I. A. (2021). Nutrients and Energy Digestibility of Microalgal Biomass for Fish Feed Applications. *Sustainability*.

[13] Bélanger A, Sarker PK, Bureau DP, Chouinard Y, Vandenberg GW (2021). Apparent Digestibility of Macronutrients and Fatty Acids from Microalgae (*Schizochytrium* sp.) Fed to Rainbow Trout (*Oncorhynchus mykiss*): A Potential Candidate for Fish Oil Substitution. *Animals*.

[14] Ganuza E., Benítez-Santana T., Atalah E., Vega-Orellana O., Ganga R., Izquierdo M. S. (2008). *Crypthecodinium cohnii* and *Schizochytrium* sp. as Potential Substitutes to Fisheries-Derived Oils from Seabream (*Sparus aurata*) Microdiets. *Aquaculture*.

[15] Santigosa E., Brambilla F., Milanese L. (2021). Microalgae Oil as an Effective Alternative Source of EPA and DHA for Gilthead Seabream (*Sparus aurata*) Aquaculture. *Animals*.

[16] Lee S., Park C. O., Choi W. (2022). Partial Substitution of Fish Oil With Microalgae (*Schizochytrium* sp.) Can Improve Growth Performance, Nonspecific Immunity and Disease Resistance in Rainbow Trout, *Oncorhynchus mykiss*. *Animals*.

[17] Neylan K. A., Johnson R. B., Barrows F. T., Marancik D. P., Hamilton S. L., Gardner L. D. (2024). Evaluating a Microalga (*Schizochytrium* sp.) As An Alternative to Fish Oil in Fish-Free Feeds for Sablefish (*Anoplopoma fimbria*). *Aquaculture*.

[18] Seong T., Uno Y., Kitagima R., Kabeya N., Haga Y., Satoh S. (2021). Microalgae as Main Ingredient for Fish Feed: Non-Fish Meal and Non-Fish Oil Diet Development for Red Sea Bream, *Pagrus Major*, by Blending of Microalgae Nannochloropsis *Chlorella* and *Schizochytrium*. *Aquaculture Research*.

[19] Karapanagiotidis I. T., Metsoviti M. N., Gkalogianni E. Z. (2022). The Effects of Replacing Fishmeal by *Chlorella vulgaris* and Fish Oil by *Schizochytrium* sp. and Microchloropsis Gaditana Blend on Growth Performance, Feed Efficiency, Muscle Fatty Acid Composition and Liver Histology of Gilthead Seabream (*Sparus aurata*). *Aquaculture*.

[20] Katsoulis-Dimitriou S., Nikouli E., Gkalogianni E. Z., Karapanagiotidis I. T., Kormas K. A. The Effect of Dietary Fish Oil Replacement by Microalgae on the Gilthead Sea Bream Midgut Bacterial Microbiota.

[21] Van Huis A., Van Itterbeeck J., Klunder H. (2013). Edible Insects: Future Prospects for Food and Feed Security. *Food and Agriculture Organization of the United Nations*.

[22] Mousavi S., Zahedinezhad S., Loh J. Y. (2020). A Review on Insect Meals in Aquaculture: The Immunomodulatory and Physiological Effects. *International Aquatic Research*.

[23] Piccolo G., Iaconisi V., Marono S. (2017). Effect of *Tenebrio molitor* Larvae Meal on Growth Performance, in Vivo Nutrients Digestibility, Somatic and Marketable Indexes of Gilthead Sea Bream (*Sparus aurata*). *Animal Feed Science and Technology*.

[24] Li Y., Kortner T. M., Chikwati E. M., Belghit I., Lock E. J., Krogdahl Å. (2020). Total Replacement of Fish Meal with Black Soldier Fly (*Hermetia illucens*) Larvae Meal Does Not Compromise the Gut Health of Atlantic Salmon (*Salmo salar*). *Aquaculture*.

[25] Mastoraki M., Katsika L., Enes P. (2022). Insect Meals in Feeds for Juvenile Gilthead Seabream (*Sparus aurata*): Effects on Growth, Blood Chemistry, Hepatic Metabolic Enzymes, Body Composition and Nutrient Utilization. *Aquaculture*.

[26] Andreadis S. S., Panteli N., Mastoraki M. (2022). Towards Functional Insect Feeds: Agri-Food by-Products Enriched with Post-Distillation Residues of Medicinal Aromatic Plants in *Tenebrio molitor* (Coleoptera: Tenebrionidae) Breeding. *Antioxidants*.

[27] Aragão C., Gonçalves A. T., Costas B., Azeredo R., Xavier M. J., Engrola S. (2022). Alternative Proteins for Fish Diets: Implications Beyond Growth. *Animals*.

[28] Henry M. A., Golomazou E., Asimaki A. (2022). Partial Dietary Fishmeal Replacement with Full-Fat or Defatted Superworm (*Zophobas Morio*) Larvae Meals Modulates the Innate Immune System of Gilthead Seabream, *Sparus aurata*. *Aquaculture Reports*.

[29] Veldkamp T., Dong L., Paul A., Govers C. (2022). Bioactive Properties of Insect Products for Monogastric Animals–A Review. *Journal of Insects as Food and Feed*.

[30] Albrektsen S., Kortet R., Skov P. V. (2022). Future Feed Resources in Sustainable Salmonid Production: A Review. *Reviews in Aquaculture*.

[31] Antonopoulou E., Panteli N., Feidantsis K. (2022). Carob (*Ceratonia siliqua*) as Functional Feed Is Beneficial in Yellow Mealworm (*Tenebrio molitor*) Rearing: Evidence from Growth, Antioxidant Status and Cellular Responses. *Antioxidants*.

[32] Ojha S., Bußler S., Schlüter O. K. (2020). Food Waste Valorisation and Circular Economy Concepts in Insect Production and Processing. *Waste Management*.

[33] Frasnetti E., Sadeqi H., Lamastra L. (2023). Integrating Insects into the Agri-Food System of Northern Italy as a Circular Economy Strategy. *Sustainable Production and Consumption*.

[34] Magalhães R., Sánchez-López A., Leal R. S., Martínez-Llorens S., Oliva-Teles A., Peres H. (2017). Black Soldier Fly (*Hermetia illucens*) Pre-Pupae Meal as a Fish Meal Replacement in Diets for European Seabass (*Dicentrarchus labrax*). *Aquaculture*.

[35] De Marco M., Martínez S., Hernandez F. (2015). Nutritional Value of Two Insect Larval Meals (*Tenebrio molitor* and *Hermetia illucens*) for Broiler Chickens: Apparent Nutrient Digestibility, Apparent Ileal Amino Acid Digestibility and Apparent Metabolizable Energy. *Animal Feed Science and Technology*.

[36] Henry M., Gasco L., Piccolo G., Fountoulaki E. (2015). Review on the Use of Insects in the Diet of Farmed Fish: Past and Future. *Animal Feed Science and Technology*.

[37] Liland N. S., Biancarosa I., Araujo P. (2017). Modulation of Nutrient Composition of Black Soldier Fly (*Hermetia illucens*) Larvae by Feeding Seaweed-Enriched Media. *PLoS One*.

[38] Belghit I., Liland N. S., Gjesdal P. (2019). Black Soldier Fly Larvae Meal Can Replace Fish Meal in Diets of Sea-Water Phase Atlantic Salmon (*Salmo salar*). *Aquaculture*.

[39] Mohan K., Rajan D. K., Muralisankar T., Ganesan A. R., Sathishkumar P., Revathi N. (2022). Use of Black Soldier Fly (*Hermetia illucens* L.) Larvae Meal in Aquafeeds for a Sustainable Aquaculture Industry: A Review of Past and Future Needs. *Aquaculture*.

[40] Panteli N., Mastoraki M., Lazarina M. (2021). Configuration of Gut Microbiota Structure and Potential Functionality in Two Teleosts Under the Influence of Dietary Insect Meals. *Microorganisms*.

[41] Di Rosa A. R., Caccamo L., Pansera L., Oteri M., Chiofalo B., Maricchiolo G. (2023). Influence of, *Hermetia illucens*, Larvae Meal Dietary Inclusion on Growth Performance, Gut Histological Traits and Stress Parameters in *Sparus aurata*. *Animals*.

[42] Pulido-Rodriguez L. F., Cardinaletti G., Secci G. (2021). Appetite Regulation, Growth Performances and Fish Quality Are Modulated by Alternative Dietary Protein Ingredients in Gilthead Gilthead Sea Bream (*Sparus aurata*) Culture. *Animals*.

[43] Fabrikov D., Vargas-García M. D. C., Barroso F. G. (2021). Effect on Intermediary Metabolism and Digestive Parameters of the High Substitution of Fishmeal with Insect Meal in *Sparus aurata* Feed. *Insects*.

[44] Carvalho M., Torrecillas S., Montero D. (2023). Insect and Single-Cell Protein Meals as Replacers of Fish Meal in Low Fish Meal and Fish Oil Diets for Gilthead Sea Bream (*Sparus aurata*) Juveniles. *Aquaculture*.

[45] Karapanagiotidis I. T., Neofytou M. C., Asimaki A. (2023). Fishmeal Replacement by Full-Fat and Defatted, *Hermetia illucens*, Prepupae Meal in the Diet of Gilthead Seabream (*Sparus aurata*). *Sustainability*.

[46] Kousoulaki K., Sveen L., Norén F., Espmark A. (2022). Atlantic Salmon (*Salmo salar*) Performance Fed Low Trophic Ingredients in a Fish Meal and Fish Oil Free Diet. *Frontiers in Physiology*.

[47] Zhao Y., Li J. (2016). *Ascidia*n Bioresources: Common and Variant Chemical Compositions and Exploitation Strategy - Examples of *Halocynthia roretzi*, *Styela plicata*, Ascidia sp. and *Ciona intestinalis*. *Zeitschrift für Naturforschung C*.

[48] Gao P., Khong H. Y., Mao W. (2023). Tunicates as Sources of High-Quality Nutrients and Bioactive Compounds for Food/Feed and Pharmaceutical Applications: A Review. *Foods*.

[49] Zhao Y., Wang M., Lindström M. E., Li J. (2015). Fatty Acid and Lipid Profiles with Emphasis on n-3 Fatty Acids and Phospholipids from *Ciona intestinalis*. *Lipids*.

[50] Samuelsen T. A., Haustveit G., Kousoulaki K. (2022). The use of Tunicate (*Ciona intestinalis*) as a Sustainable Protein Source in Fish Feed – Effects on the Extrusion Process, Physical Pellet Quality and Microstructure. *Animal Feed Science and Technology*.

[51] Fantatto R. R., Mota J., Ligeiro C. (2024). Exploring Sustainable Alternatives in Aquaculture Feeding: The Role of Insects. *Aquaculture Reports*.

[52] Sarker P. K. (2023). Microorganisms in Fish Feeds, Technological Innovations, and Key Strategies for Sustainable Aquaculture. *Microorganisms*.

[53] Carvalho M., Ginés R., Martín I. (2024). Genetic Selection for High Growth Improves the Efficiency of Gilthead Sea Bream (*Sparus aurata*) in Using Novel Diets with Insect Meal, Single-Cell Protein and a DHA Rich-Microalgal Oil. *Aquaculture*.

[54] Iqbal M., Yaqub A., Ayub M. (2022). Partial and Full Substitution of Fish Meal and Soybean Meal by Canola Meal in Diets for Genetically Improved Farmed Tilapia (O. *Niloticus*): Growth Performance, Carcass Composition, Serum Biochemistry, Immune Response, and Intestine Histology. *Journal of Applied Aquaculture*.

[55] Gómez-Requeni P., Mingarro M., Calduch-Giner J. A. (2004). Protein Growth Performance, Amino Acid Utilisation and Somatotropic Axis Responsiveness to Fish Meal Replacement by Plant Protein Sources in Gilthead Sea Bream (*Sparus aurata*). *Aquaculture*.

[56] Santigosa E., García-Meilán I., Valentin J. M. (2011). Modifications of Intestinal Nutrient Absorption in Response to Dietary Fish Meal Replacement by Plant Protein Sources in Sea Bream (*Sparus aurata*) and Rainbow Trout (*Onchorynchus Mykiss*). *Aquaculture [Internet]*.

[57] Mente E., Bousdras T., Feidantsis K. (2022). *Tenebrio molitor* Larvae Meal Inclusion Affects Hepatic Proteome and Apoptosis and/or Autophagy of Three Farmed Fish Species. *Scientific Reports*.

[58] Moutinho S., Peres H., Serra C. (2017). Meat and Bone Meal as Partial Replacement of Fishmeal in Diets for Gilthead Sea Bream (*Sparus aurata*) Juveniles: Diets Digestibility, Digestive Function, and Microbiota Modulation. *Aquaculture*.

[59] Antonopoulou E., Nikouli E., Piccolo G. (2019). Reshaping Gut Bacterial Communities after Dietary *Tenebrio molitor* Larvae Meal Supplementation in Three Fish Species. *Aquaculture*.

[60] Feidantsis K., Kaitetzidou E., Mavrogiannis N., Michaelidis B., Kotzamanis Y., Antonopoulou E. (2014). Effect of Taurine-Enriched Diets on the Hsp Expression, MAPK Activation and the Antioxidant Defence of the European Sea Bass (*Dicentrarchus labrax*). *Aquaculture Nutrition*.

[61] Hotamisligil G. S., Davis R. J. (2016). Cell Signaling and Stress Responses. *Cold Spring Harbor Perspectives in Biology*.

[62] Antonopoulou E., Chouri E., Feidantsis K., Lazou A., Chatzifotis S. (2017). Effects of Partial Dietary Supplementation of Fish Meal with Soymeal on the Stress and Apoptosis Response in the Digestive System of Common Dentex (*Dentex dentex*). *Journal of Biological Research-Thessaloniki*.

[63] Li S., Ji H., Zhang B., Zhou J., Yu H. (2017). Defatted Black Soldier Fly (*Hermetia illucens*) Larvae Meal in Diets for Juvenile Jian Carp (*Cyprinus carpio* Var. Jian): Growth Performance, Antioxidant Enzyme Activities, Digestive Enzyme Activities, Intestine and Hepatopancreas Histological Structure. *Aquaculture*.

[64] Karl P. J., Hatch A. M., Arcidiacono S. M. (2018). Effects of Psychological, Environmental and Physical Stressors on the Gut Microbiota. *Frontiers in Microbiology*.

[65] Ye Z., Xu Y. J., Liu Y. (2021). Influence of Different Dietary Oil Consumption on Nutrient Malabsorption: An Animal Trial Using Sprague Dawley Rats. *Journal of Food Biochemistry*.

[66] Rimoldi S., Antonini M., Gasco L., Moroni F., Terova G. (2021). Intestinal Microbial Communities of Rainbow Trout (*Oncorhynchus mykiss*) May Be Improved by Feeding a *Hermetia illucens* Meal/Low-Fishmeal Diet. *Fish Physiology and Biochemistry*.

[67] han Yu M., shan Li X., Wang J. (2023). Substituting Fish Meal With a Bacteria Protein (*Methylococcus capsulatus*, Bath) Grown on Natural Gas: Effects on Growth Non-Specific Immunity and Gut Health of Spotted Seabass (*Lateolabrax Maculatus*). *Animal Feed Science and Technology*.

[68] Panteli N., Kousoulaki K., Antonopoulou E. (2024). Which Novel Ingredient Should Be Considered the, Holy Grail, for Sustainable Production of Finfish Aquafeeds?. *Reviews in Aquaculture*.

[69] Kroeckel S., Harjes A. G. E., Roth I. (2012). When a Turbot Catches a Fly: Evaluation of a Pre-Pupae Meal of the Black Soldier Fly (*Hermetia illucens*) as Fish Meal Substitute - Growth Performance and Chitin Degradation in Juvenile Turbot (*Psetta Maxima*). *Aquaculture*.

[70] Randazzo B., Zarantoniello M., Cardinaletti G. (2021). *Hermetia illucens* and Poultry by-Product Meals as Alternatives to Plant Protein Sources in Gilthead Seabream (*Sparus aurata*) Diet: A Multidisciplinary Study on Fish Gut Status. *Animals*.

[71] Tampou A., Andreopoulou S., Vasilaki A. (2024). Growth Performance of Gilthead Sea Bream (*Sparus aurata*) Fed a Mixture of Single Cell Ingredients for Organic Diets. *Aquaculture Reports*.

[72] Lyndonl A. R., Davidson I., Houlihan D. F. (1993). Changes in Tissue and Plasma Free Amino Acid Concentrations after Feeding in Atlantic Cod. *Fish Physiology and Biochemistry*.

[73] Mente E., Coutteau P., Houlihan D., Davidson I., Sorgeloos P. (2002). Protein Turnover, Amino Acid Profile and Amino Acid Flux in Juvenile Shrimp *Litopenaeus vannamei*: Effects of Dietary Protein Source. *Journal of Experimental Biology*.

[74] Arai S. (1981). A Purified Test Diet for Coho Salmon, *Oncorhynchus kisutch*, Fry. *Nippon Suisan Gakkaishi*.

[75] Fountoulaki E., Alexis M. N., Nengas I., Venou B. (2003). Effects of Dietary Arachidonic Acid (20: 4n-6), on Growth, Body Composition, and Tissue Fatty Acid Profile of Gilthead Bream Fingerlings (*Sparus aurata* L.). *Aquaculture*.

[76] Driedzic W. R., De Almeida-Val V. M. F. (1996). Enzymes of Cardiac Energy Metabolism in Amazonian Teleosts and the Fresh-Water Stingray (*Potamotrygon hystrix*). *The Journal of Experimental Zoology*.

[77] Moon T. W., Mommsen T. P. (1987). Enzymes of Intermediary Metabolism in Tissue of the Little Skate, *Raja Erinacea*. *Journal of Experimental Zoology*.

[78] Sidell B. D., Driedzic W. R., Stowe D. B., Johnston I. A. (1987). Biochemical Correlations of Power Development and Metabolic Fuel Preferenda in Fish Hearts. *Physiological Zoology*.

[79] Singer T. D., Ballantyne J. S. (1989). Absence of Extrahepatic Lipid Oxidation in a Freshwater Elasmobranch, the Dwarf Stingray *Potamotrygon magdalenae*: Evidence from Enzyme Activities. *Journal of Experimental Zoology*.

[80] Antonopoulou E., Kousidou E., Tserga E., Feidantsis K., Chatzifotis S. (2014). Dietary Lipid Levels in Meagre (*Argyrosomus regius*): Effects on Biochemical and Molecular Indicators of Liver. *Aquaculture*.

[81] Moutinho S., Oliva-Teles A., Martínez-Llorens S., Monroig Ó., Peres H. (2022). Total Fishmeal Replacement by Defatted *Hermetia illucens* Larvae Meal in Diets for Gilthead Seabream (*Sparus aurata*) Juveniles. *Journal of Insects as Food and Feed*.

[82] Mastoraki M., Panteli N., Kotzamanis Y. P., Gasco L., Antonopoulou E., Chatzifotis S. (2022). Nutrient Digestibility of Diets Containing Five Different Insect Meals in Gilthead Sea Bream (*Sparus aurata*) and European Sea Bass (*Dicentrarchus labrax*). *Animal Feed Science and Technology*.

[83] Gasco L., Józefiak A., Henry M. (2021). Beyond the Protein Concept: Health Aspects of Using Edible Insects on Animals. *Journal of Insects as Food and Feed*.

[84] Osimani A., Milanović V., Roncolini A. (2019). *Hermetia illucens* in Diets for Zebrafish (*Danio rerio*): A Study of Bacterial Diversity by Using PCR-DGGE and Metagenomic Sequencing. *PLoS One*.

[85] Zarantoniello M., Zimbelli A., Randazzo B. (2020). Black Soldier Fly (*Hermetia illucens*) Reared on Roasted Coffee by-Product and *Schizochytrium* sp. as a Sustainable Terrestrial Ingredient for Aquafeeds Production. *Aquaculture*.

[86] Ribeiro A. R., Gonçalves A., Barbeiro M. (2017). *Phaeodactylum tricornutum* in Finishing Diets for Gilthead Seabream: Effects on Skin Pigmentation, Sensory Properties and Nutritional Value. *Journal of Applied Phycology*.

[87] Reis B., Ramos-Pinto L., Martos-Sitcha J. A. (2021). Health Status in Gilthead Seabream (*Sparus aurata*) Juveniles Fed Diets Devoid of Fishmeal and Supplemented With *Phaeodactylum tricornutum*. *Journal of Applied Phycology*.

[88] Carvalho M., Marotta B., Xu H. (2022). Complete Replacement of Fish Oil by Three Microalgal Products Rich in n-3 Long-Chain Polyunsaturated Fatty Acids in Early Weaning Microdiets for Gilthead Sea Bream (*Sparus aurata*). *Aquaculture*.

[89] Eryalçin K. M., Yildiz M. (2015). Effects of Long-Term Feeding With Dried Microalgae Added Microdiets on Growth and Fatty Acid Composition on Gilthead Gilthead Sea Bream (*Sparus aurata* L., 1758). *Turkish Journal of Fisheries and Aquatic Sciences*.

[90] Ferreira M., Ribeiro P. C., Ribeiro L. (2022). Effects on Growth Performance, Nutrient Utilization, and Tissue Composition of Gilthead Seabream (*Sparus aurata*). *Frontiers in Physiology*.

[91] Adamidou S., Nengas I., Henry M. (2011). Effects of Dietary Inclusion of Peas, Chickpeas and Faba Beans on Growth, Feed Utilization and Health of Gilthead Seabream (*Sparus aurata*). *Aquaculture Nutrition*.

[92] Adamidou S., Nengas I., Alexis M. (2009). Apparent Nutrient Digestibility and Gastrointestinal Evacuation Time in European Seabass (*Dicentrarchus labrax*) Fed Diets Containing Different Levels of Legumes. *Aquaculture*.

[93] Adamidou S., Nengas I., Henry M. (2009). Growth, Feed Utilization, Health and Organoleptic Characteristics of European Seabass (*Dicentrarchus labrax*) Fed Extruded Diets including Low and High Levels of Three Different Legumes. *Aquaculture*.

[94] Pulido L., Secci G., Maricchiolo G. (2022). Effect of Dietary Black Soldier Fly Larvae Meal on Fatty Acid Composition of Lipids and Sn-2 Position of Triglycerides of Marketable Size Gilthead Sea Bream Fillets. *Aquaculture*.

[95] Kousoulaki K., Carlehög M., Mørkøre T., Ruyter B., Berge G. M. (2017). Long Term Supplementation of Heterotrophic Microalgae in Atlantic Salmon Diets. *Aquaculture Europe*.

[96] Perera E., Sánchez-Ruiz D., Sáez M. I. (2020). Low Dietary Inclusion of Nutraceuticals From Microalgae Improves Feed Efficiency and Modifies Intermediary Metabolisms in Gilthead Sea Bream (*Sparus aurata*). *Scientific Reports*.

[97] Bousdras T., Feidantsis K., Panteli N. (2022). Larvae Meal Inclusion Exerts Tissue-Specific Effects on Cellular, Metabolic, and Antioxidant Status in European Sea Bass (*Dicentrarchus labrax*) and Gilthead Seabream (*Sparus aurata*). *Aquaculture Nutrition*.

[98] Moura C. S., Lollo P. C. B., Morato P. N., Amaya-Farfan J. (2018). Dietary Nutrients and Bioactive Substances Modulate Heat Shock Protein (HSP) Expression: A Review. *Nutrients*.

[99] Feidantsis K., Pörtner H. O., Markou T., Lazou A., Michaelidis B. (2012). Involvement of p38 MAPK in the Induction of Hsp70 During Acute Thermal Stress in Red Blood Cells of the Gilthead Sea Bream, *Sparus aurata*. *Journal of Experimental Zoology Part A: Ecological Genetics and Physiology*.

[100] Antonopoulou E., Kolygas M., Panteli N. (2023). Breeding Substrate Containing Distillation Residues of Mediterranean Medicinal Aromatic Plants Modulates the Effects of *Tenebrio molitor* as Fishmeal Substitute on Blood Signal Transduction and WBC Activation of Gilthead Seabream (*Sparus aurata*). *Animals*.

[101] Freeman-Cook K. D., Autry C., Borzillo G. (2010). Design of Selective, ATP-Competitive Inhibitors of Akt. *Journal of Medicinal Chemistry*.

[102] Liu Y., Chang H., Lv W. (2022). Physiological Response of Rainbow Trout (*Oncorhynchus mykiss*) to Graded Levels of Novel *Chlorella Sorokiniana* Meal as a Single Fishmeal Alternative or Combined With Black Soldier Fly Larval Meal. *Aquaculture*.

[103] Winder W. A., Hardie D. G. (1999). AMP-Activated Protein Kinase, a Metabolic Master Switch: Possible Roles in Type 2 Diabetes. *American Journal of Physiology*.

[104] Piazzon M. C., Naya-Català F., Pereira G.V. (2022). A Novel Fish Meal-Free Diet Formulation Supports Proper Growth and Does Not Impair Intestinal Parasite Susceptibility in Gilthead Sea Bream (*Sparus aurata*) With a Reshape of Gut Microbiota and Tissue-Specific Gene Expression Patterns. *Aquaculture*.

[105] Morrison D. J., Preston T. (2016). Formation of Short Chain Fatty Acids by the Gut Microbiota and Their Impact on Human Metabolism. *Gut Microbes*.

[106] Høgsgaard K., Vidal N. P., Marietou A. (2023). Fucose Modifies Short Chain Fatty Acid and H_2_S Formation Through Alterations of Microbial Cross-Feeding Activities. *FEMS Microbiology Ecology*.

[107] Fusco W., Lorenzo M. B., Cintoni M. (2023). Short-Chain Fatty-Acid-Producing Bacteria: Key Components of the Human Gut Microbiota. *Nutrients*.

[108] Ray A. K., Ghosh K., Ringø E. (2012). Enzyme-Producing Bacteria Isolated From Fish Gut: A Review. *Aquaculture Nutrition*.

[109] Askarian F., Zhou Z., Olsen R. E., Sperstad S., Ringø E. (2012). Culturable Autochthonous Gut Bacteria in Atlantic Salmon (*Salmo salar* L.) Fed Diets With or Without Chitin. Characterization by 16S rRNA Gene Sequencing, Ability to Produce Enzymes and in Vitro Growth Inhibition of Four Fish Pathogens. *Aquaculture*.

[110] MacDonald N. L., Stark J. R., Austin B. (1986). Bacterial Microflora in the Gastro-Intestinal Tract of Dover Sole (*Solea solea* L.), With Emphasis on the Possible Role of Bacteria in the Nutrition of the Host. *FEMS Microbiology Letters*.

[111] Skrodenyte-Arbačiauskiene V. (2007). Enzymatic Activity of Intestinal Bacteria in Roach, *Rutilus rutilus*, L. *Fisheries Science*.

[112] Gupta S., Fečkaninová A., Lokesh J. (2019). Lactobacillus Dominate in the Intestine of Atlantic Salmon Fed Dietary Probiotics. *Frontiers in Microbiology*.

[113] Garibay-Valdez E., Cicala F., Martinez-Porchas M. (2021). Longitudinal Variations in the Gastrointestinal Microbiome of the White Shrimp, *Litopenaeus vannamei*. *Peer J*.

[114] Albertsen M., Hugenholtz P., Skarshewski A., Nielsen K. L., Tyson G. W., Nielsen P. H. (2013). Genome Sequences of Rare, Uncultured Bacteria Obtained by Differential Coverage Binning of Multiple Metagenomes. *Nature Biotechnology*.

[115] Eichmiller J. J., Hamilton M. J., Staley C., Sadowsky M. J., Sorensen P. W. (2016). Environment Shapes the Fecal Microbiome of Invasive Carp Species. *Microbiome*.

[116] Cheng Y., Ge C., Li W., Yao H. (2021). The Intestinal Bacterial Community and Functional Potential of *Litopenaeus vannamei* in the Coastal Areas of China. *Microorganisms*.

[117] Zárate G., ed.E.Cid Dairy Propionibacteria: less Conventional Probiotics to Improve the Human and Animal Health. *Probiotic in Animals*.

[118] Rogall E. T., Jacob S., Triebskorn R., Schwartz T. (2020). The Impact of the Anti-Diabetic Drug Metformin on the Intestinal Microbiome of Larval Brown Trout (*Salmo trutta* f. *Fario*). *Environmental Sciences Europe*.

[119] Říhová J., Batani G., Rodríguez-Ruano S. M. (2021). A New Symbiotic Lineage Related to Neisseria and Snodgrassella Arises From the Dynamic and Diverse Microbiomes in Sucking Lice. *Molecular Ecology*.

[120] Sehnal L., Brammer-Robbins E., Wormington A. M. (2021). Microbiome Composition and Function in Aquatic Vertebrates. Small Organisms Making Big Impacts on Aquatic Animal Health. *Frontiers in Microbiology*.

[121] Luan Y., Li M., Zhou W. (2023). The Fish Microbiota: Research Progress and Potential Applications. *Engineering*.

[122] Zhang C., Yu Z., Xue Z. (2021). The Temporal Dynamics of Bacteria in the Coelomic Fluid of Sea Cucumber *Apostichopus japonicus* After Evisceration. *Invertebrate Survival Journal*.

[123] Abd El-Rhman A. M., Khattab YAE., Shalaby AME. (2009). *Micrococcus luteus* and *Pseudomonas* Species as Probiotics for Promoting the Growth Performance and Health of Nile Tilapia, *Oreochromis niloticus*. *Fish & Shellfish Immunology*.

[124] Liu Q., Akbar S., Ding Z. Induced Anti-Predation Defense Traits of Daphnia Are Associated With the Gut Microbiota Composition.

[125] Liu C., Zhao L. P., Shen Y. Q. (2021). A Systematic Review of Advances in Intestinal Microflora of Fish. *Fish Physiology and Biochemistry*.

[126] Veckman V., Miettinen M., Matikainen S. (2003). *Lactobacilli* and *Streptococci* Induce Inflammatory Chemokine Production in Human Macrophages that Stimulates Th1 Cell Chemotaxis. *Journal of Leukocyte Biology*.

[127] Nguyen C. D. H., Amoroso G., Ventura T., Minich J. J., Elizur A. (2020). Atlantic Salmon (*Salmo salarL.*, 1758) Gut Microbiota Profile Correlates with Flesh Pigmentation: Cause or Effect?. *Marine Biotechnology*.

[128] Cua L. S., Stein L. Y. (2014). Characterization of Denitrifying Activity by the Alphaproteobacterium, *Sphingomonas wittichii*, RW1. *Frontiers in Microbiology*.

[129] Wu M., Huang H., Li G. (2017). The Evolutionary Life Cycle of the Polysaccharide Biosynthetic Gene Cluster Based on the Sphingomonadaceae. *Scientific Reports*.

[130] Nan Q., Wang C., Wang H., Yi Q., Wu W. (2020). Mitigating Methane Emission via Annual Biochar Amendment Pyrolyzed With Rice Straw From the Same Paddy Field. *Science of The Total Environment*.

[131] Rosenberg E., Rosenberg E., DeLong E. F., Lory S., Stackebrandt E., Thompson F. (2014). The Family Chitinophagaceae. *The Prokaryotes: Other Major Lineages of Bacteria and the Archaea*.

[132] Givens C. E., Ransom B., Bano N., Hollibaugh J. T. (2015). Comparison of the Gut Microbiomes of 12 Bony Fish and 3 shark Species. *Marine Ecology Progress Series*.

[133] Bruni L., Pastorelli R., Viti C., Gasco L., Parisi G. (2018). Characterisation of the Intestinal Microbial Communities of Rainbow Trout (*Oncorhynchus mykiss*) Fed With *Hermetia illucens* (Black Soldier Fly) Partially Defatted Larva Meal as Partial Dietary Protein Source. *Aquaculture*.

